# Development of a sink–source interaction model for the growth of short-rotation coppice willow and *in silico* exploration of genotype×environment effects

**DOI:** 10.1093/jxb/erv507

**Published:** 2015-12-10

**Authors:** M. Cerasuolo, G. M. Richter, B. Richard, J. Cunniff, S. Girbau, I. Shield, S Purdy, A. Karp

**Affiliations:** ^1^Sustainable Soils and Grassland Systems Department, Rothamsted Research, Harpenden, Herts AL5 2JQ, UK; ^2^Agroecology Department, Rothamsted Research, Harpenden, Herts AL5 2JQ, UK; ^3^Institute of Biological Environmental and Rural Sciences (IBERS), Aberystwyth University, Plas Gogerddan, Aberystwyth, Ceredigion SY23 3EE, UK

**Keywords:** Carbon allocation, genotype, modelling, Salix, sensitivity analysis, sink–source interaction.

## Abstract

Identifying key performance traits is essential for elucidating crop growth processes and breeding. In Salix spp., genotypic diversity is being exploited to tailor new varieties to overcome environmental yield constraints. Process-based models can assist these efforts by identifying key parameters of yield formation for different genotype×environment (G×E) combinations. Here, four commercial willow varieties grown in contrasting environments (west and south-east UK) were intensively sampled for growth traits over two 2-year rotations. A sink–source interaction model was developed to parameterize the balance of source (carbon capture/mobilization) and sink formation (morphogenesis, carbon allocation) during growth. Global sensitivity analysis consistently identified day length for the onset of stem elongation as most important factor for yield formation, followed by various ‘sink>source’ controlling parameters. In coastal climates, the chilling control of budburst ranked higher compared with the more eastern climate. Sensitivity to drought, including canopy size and rooting depth, was potentially growth limiting in the south-east and west of the UK. Potential yields increased from the first to the second rotation, but less so for broad- than for narrow-leaved varieties (20 and 47%, respectively), which had established less well initially (–19%). The establishment was confounded by drought during the first rotation, affecting broad- more than narrow-leaved canopy phenotypes (–29%). The analysis emphasized quantum efficiency at low light intensity as key to assimilation; however, on average, sink parameters were more important than source parameters. The G×E pairings described with this new process model will help to identify parameters of sink–source control for future willow breeding.

## Highlight

The process-based model LUCASS gave insights into the sink–source control of willow growth, identifying key parameters and predicting the performance of contrasting canopy phenotypes in different environments.

## Introduction

Yield improvement is an important objective in the development of woody biomass feedstocks from short-rotation coppice (SRC) (Volk *et al.*, 2006; Karp and Shield, 2008). Poplar (Populus spp.) and willow (Salix spp.) are comparatively young in their domestication (Karp *et al.*, 2011; Stanton *et al.*, 2014) and pedo-climatic adaptation (Aylott *et al.*, 2008; Toillon *et al.*, 2013). However, yields have been doubled by breeders selecting mainly for total harvestable biomass, stem traits, and disease resistance (Karp *et al.*, 2011). Traits associated with vigour (Kauter *et al.*, 2003; Weih and Nordh, 2005; Volk *et al.*, 2006; Verlinden *et al.*, 2013), development (Verwijst *et al.*, 2012; Toillon *et al.*, 2013), photosynthesis (Robinson *et al.*, 2004; Andralojc *et al.*, 2014), and water-use efficiency (Weih and Nordh, 2002; Deckmyn *et al.*, 2004) are now also being incorporated. However, experimental evidence for carbon assimilation (source formation) and its linkage to allocation (sink formation) to above-ground biomass (AGB) (Bullard *et al.*, 2002b; Laureysens *et al.*, 2003; Guidi *et al.*, 2009) and below-ground biomass (BGB) (Heilman *et al.*, 1994; Matthews, 2001; Rytter, 2001; Martin and Stephens, 2006; Pacaldo *et al.*, 2013) has yet to be integrated.

Process-based simulation models are useful tools for integrating knowledge and assessing the relative importance of traits, particularly in woody perennials with long growth cycles, where gathering experimental data is time consuming and expensive (Karp *et al.*, 2014). SRC comprises successive harvest (coppicing) rotations lasting 2–3 years, where ‘regrowth’ after harvest occurs from basal buds on coppiced stools, while successive ‘annual growth’ before harvest occurs from buds on the stems. Annual regrowth from reserves was poorly addressed in early models (Le Roux *et al.*, 2001), despite its importance (Ceulemans *et al.*, 1996; Philippot, 1996). The existing models for SRC treat growth either as source dependent (‘top down’), limited by light interception and use efficiency (Tharakan *et al.*, 2008), or as sink dependent (‘bottom up’) and influenced by coppice response and carbon allocation (Deckmyn *et al.*, 2004). For phenological development, a simple empirical canopy model (Deckmyn *et al.*, 2004) was later replaced by a temperature-controlled budburst model (Deckmyn *et al.*, 2008), and adapted for SRC without validation (Tallis *et al.*, 2013). The need to model temperature control of dormancy, currently debated in many species (Fu *et al.*, 2012), is also unclear in willow (Savage and Cavender-Bares, 2011). Indeed, the whole system of yield formation, dormancy break, budburst, and start of photosynthesis needs to be integrated with the initiation of stem growth (sink formation).

Carbon allocation and sink formation have often been simplified in previous models (Deckmyn *et al.*, 2004) and allocation to BGB reserves ignored, despite its potential importance for regrowth after coppicing (Tschaplinski and Blake, 1995; Ceulemans *et al.*, 1996). Control of early soft-wood production by labile carbon allocated to tree reserves (Deckmyn *et al.*, 2008) has recently been observed for SRC (Verwijst *et al.*, 2012). The importance of reserves for regrowth was also shown in perennial forage crops (Schapendonk *et al.*, 1998; Teixeira *et al.*, 2007). For grassland, a sink–source interaction model was proposed (Schapendonk *et al.*, 1998) where assimilate allocation is controlled by sink formation. A similar control has been implemented for carbon partitioning in forest models (Fourcaud *et al.*, 2008; Pretzsch *et al.*, 2008). Carbon translocation is important for both SRC and grass, which show die-back of stems and tillers, respectively. Data from empirical protocols (stem number, length, and diameter), used for SRC phenotyping, can be used for the development of a hybrid model, which combines morphometric data with an eco-physiological process model as suggested by Pretzsch *et al.* (2008). This is similar to the approach to predict yields of specific willow clones proposed by Amichev *et al.* (2011).

To simulate growth processes sufficiently well for use in willow breeding, there is a clear need to derive an integrated model that adequately incorporates key phenological processes and morphogenesis controlling AGB, while also taking into account BGB and the influence of reserves. In particular, it is important to integrate new experimental evidence, assess sink and source limitations, rank genotype-specific parameters, and identify the most important ones to focus breeding efforts.

To address these challenges, we developed a sink–source interaction model, LUCASS (light use and carbon allocation in Salix species) in which phenology controls growth and yield formation. This model describes and predicts the growth of four commercial willow genotypes. The model was calibrated for potential and water-limited production using detailed field data at two different sites in the UK. Key parameters for yield formation, across varieties and in different environments, were identified using a global sensitivity analysis (SA), including all parameters. Finally, the model was validated against independent growth and yield datasets.

## Materials and methods

### Field experiments

Detailed observations describing plant growth were recorded in two identical field trials laid out in a randomized block design consisting of four blocks (Cunniff *et al.*, 2015). Each block contained four commercial Salix varieties: Endurance (*Salix redheriana*×*Salix dasyclados*), Resolution (multiple parental crosses of *Salix viminalis*×*Salix schwerinii*), Terra Nova [(*S. viminalis*×*Salix triandra*)×*Salix miyabeana*], and Tora [*S. schwerinii*×(*S. viminalis*×*S. viminalis*)] in individual plots of 224 m^2^. Details can be found in Cunniff *et al.* (2015, see Table 1). These varieties were grouped according to growth and canopy phenotype (see Supplementary Fig. S1 at *JXB* online) into broad-leaved (20–27mm), closed canopy (Endurance, Terra Nova) and narrow-leaved (14–19mm), open canopy (Resolution, Tora). The crops were planted in double rows (16 000 cuttings ha^–1^) in May 2009 and coppiced in January 2010, 2012, and 2014. Destructive and non-destructive measurements of AGB and BGB traits were taken from respective plot areas during two successive 2-year rotations (R1, first rotation, 2010–2011; R2, second rotation, 2012–2013) to populate an extensive database for research and model development.

**Table 1.** Meteorological indicators during dormancy (November–March) and growth (April–October) periods Mean maximal and minimal air temperatures (*T*_max_ and *T*_min_, respectively) and cumulative annual global radiation (*R*_g_) and precipitation (*P*) were recorded at the three sites.

Site	Dormancy		Growth		*R*_g_ (MJ m^–2^)	*P* (mm)
	*T*_max_ (°C)	*T*_min_ (°C)	*T*_max_ (°C)	T_min_ (°C)		
ROTH	7.6	1.8	17.7	9.0	3910	680
ABER	9.0	3.8	16.6	11.6	3560	1020
LARS	9.2	3.1	18.5	10.4	3740	760

#### Locations 

The experiments used for model parameterization (where previous data were not available), calibration, and internal evaluation were located in south-east England (51.82° N, 0.38° W) at Rothamsted (ROTH) and Aberystwyth (ABER) in Wales (52.4139° N, 4.014° W). Soils were characterized as a silty clay loam (chromic Luvisol; 1 m depth) and a shallow sandy silt loam (eutric endoleptic Cambisol; 0.55 m depth), respectively. Harvested yields were available for three of the four varieties, which were collected from successive 2- and 3-year rotations from separate trials at Long Ashton (LARS) in Somerset, England (51.43° N, 2.65° W) and ROTH between 2001 and 2010, were used for external model validation. LARS soil was classified as a coarse loam over clay (stagnogley) to clay (argillic Pelosol) (see Supplementary Table S1 at *JXB* online).

The long-term averages characterize ROTH as drier with a higher probability of water stress (WS) (704mm, 9.3 °C) than ABER (1038mm, 9.7 °C). Site-specific hourly weather data were recorded. The first rotation (R1, 2010–2011) was drier, while the second rotation (R2), especially 2012, was wetter than the long-term average (see Supplementary Fig. S2 and Supplementary Table S2 at *JXB* online). Over all years, annual radiation at ROTH was about 20% higher and the temperature range (*T*_min_/*T*_max_) wider than at ABER (Table 1).

#### Phenotyping

Budburst was recorded annually (2010–2013), and buds were scored on 10 trees from early February twice weekly until bud swelling and then checked daily. Adapting a 7-point scale (Weih, 2009), budburst was defined as green leaf tips (<5mm) being visible. Senescence was scored weekly from September to October using 10 trees per treatment and block, adapting a 7-point scale (Fracheboud *et al.*, 2009), defining its onset as >25% yellow/brown and <10% abscised leaves.

Plant architecture (height, stem length and diameter, and number of stems) was assessed on two pairs of trees, randomly chosen from the non-destructive area of each plot. Leaf area indices (LAIs) were estimated at ROTH twice monthly using the SunScan Canopy Analysis system (Delta-T Devices Ltd, Cambridge, UK); for details see Cerasuolo *et al.* (2013). Light-use efficiency (LUE) was estimated from simulated cumulative woody stem biomass and absorbed photosynthetic active radiation (APAR) based on calibrated LAIs.

Carbon allocation rates to AGB and BGB components were determined during the first rotation (2010–2012) by destructively sampling two complete trees per plot at key phenological stages (Cunniff *et al.*, 2015). Stool (including remnant cut stem) and roots were excavated to a depth of 0.3 m, which is likely to represent >90% of the BGB (Pacaldo *et al.*, 2013). Destructive measurements of leaf weight and area were recorded (Cerasuolo *et al.*, 2013). During the second rotation (2012–2013), the number of destructive samples was reduced to twice a year, and final yields were assessed after each 2-year coppicing cycle (Cunniff *et al.*, 2015).

### Model description

**Fig. 1.**
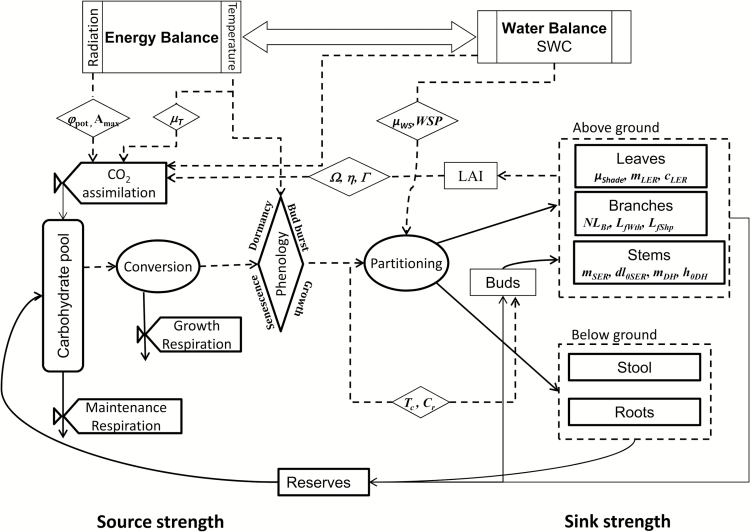
Flowchart of the process-based willow growth model LUCASS, embedded into a water and energy balance framework. See text for details.

The process-based willow growth model LUCASS (Fig. 1) simulates development and growth of Salix spp. at the stand scale, considering phenological (budburst, growth, senescence, and dormancy) and morphological plant development (sink formation), and light interception, photosynthesis, and respiration (source formation). The AGB organs (leaves, branches, and stems) and BGB organs (stool and all roots) are considered as sinks, and the carbon allocation to these sinks is phenologically controlled and balanced within the sink–source interaction model (Schapendonk *et al.*, 1998). The sinks are phenotypically dimensioned by stem and leaf numbers, their respective elongation rates, and specific dry matter densities, which define the carbon demand from a common source pool fuelled by photosynthesis and mobilizable reserves.

These processes are controlled by external variables (global radiation, air temperature, and water availability), provided by an environmental modelling framework (Richter *et al.*, 2006, 2010) that simulates the water and energy balance. LUCASS follows a bottom-up approach where light interception, photosynthesis, and respiration (Goudriaan and van Laar, 1994) are simulated with an hourly time step as part of the energy balance. Assimilate allocation to biomass components (leaf, branch, stem, stool, and roots) and respective reserve pools are calculated daily. Source–sink carbon flows are considered independently; however, carbon from senescing biomass (leaves, branch die-back, and fine roots) is translocated to the reserves.

#### Phenology 

LUCASS simulates the multi-annual cycle of phenological development at the centre of process control (Fig. 1): budburst and leaf emergence, growth of individual organs, senescence and stem die-back, and dormancy to control the onset and duration of carbon capture (source formation) and its allocation to various sinks, as has been done in grape vine (Vivin *et al.*, 2002).

##### Budburst, leaf emergence, and elongation

Similar to earlier work (Tallis *et al.*, 2013), the budburst was simulated by combining a chilling phase followed by a forcing period (Chuine, 2000; Hlaszny *et al.*, 2012). Budburst dates were calculated (Eq. S1 at *JXB* online) using daily mean air temperature (*T*_avr_), a half-efficiency temperature (*T*_C_) and a chilling threshold (*C*_r_); both *T*_C_ and *C*_r_ were estimated using genotype-specific budburst data. The other parameters defining the temperature response curves were adapted from Hlaszny *et al.* (2012). Chilling unit accumulation started with senescence during the previous season. In keeping with the known biology (Rinne *et al.*, 2011), negative chill units (*C*_u_) accumulate during endodormancy until plants reach *C*_r_ (Cesaraccio *et al.*, 2004). At this point, the model plants enter ecodormancy, an inactive ‘standby’ phase, to accumulate daily forcing (anti-chill) units (*C*_a_), which results in budburst when *C*_r_ *+∑C*_a_ *≥*0.

Leaf emergence rate was calculated as suggested by Porter *et al.* (1993) and adjusted by photoperiod, water availability, and level of reserves (Eq. S2 at *JXB* online). The leaf emergence declined exponentially over the year. The potential leaf elongation rate was considered to be dependent on average temperature and day length (Mcdonald and Stadenberg, 1993), modified for plant age (Robinson *et al.*, 2004) and WS (Eq. S3 at *JXB* online).

##### Senescence and canopy duration

The model considers leaf senescence as a function of age (accumulated thermal time, *μ*_T_), shading (*μ*_Shade_, LAI >3), and WS (*μ*__WS__). The start of senescence depends on a threshold day length, while the date of growth cessation (budset) is modelled as a function of accumulated thermal time; both values were estimated using experimental data (senescence score; end of stem extension) collected at ROTH and ABER during R1.

##### Stem and woody biomass development

Experimental evidence suggested modelling the onset and rate of stem extension as a function of day length (Eq. S5 at *JXB* online) with a developmental switch considering the base temperature for stem elongation (*T*_bStE_ =10 °C). This is in contrast to grass models in which leaf and stem extension are determined by temperature (Schapendonk *et al.*, 1998; Hoeglind *et al.*, 2005). The dynamics of stem number is described by a function of the number of initial buds that form stems and their calibrated die-back rate. The demography of leaves (Porter *et al.*, 1993) and stems (Schapendonk *et al.*, 1998; Hoeglind *et al.*, 2005) was incorporated in order to consider the empirical evidence for regrowth after coppicing, e.g. die-back (self-thinning) of stems. The model does not consider plant mortality (Bullard *et al.*, 2002b) but rather plant density.

#### Sink formation 

##### Leaf area and biomass

Total leaf area is a function of leaf number, size, and shape, scaled to LAI (Eq. S4 at *JXB* online). Initially, willow varieties produce a large number of small shoots in order to rapidly increase leaf area. These branches were treated as ‘super-leaves’ (long leaves whose area is equal to the cumulative area of leaves on the branch), whose growth rate follows the normal leaf emergence and elongation rate. The leaf area is converted to leaf biomass using a dynamic specific leaf area (SLA_min/max_; Schapendonk *et al.*, 1998) that accounts for observed variable leaf weight and area ratios (Cunniff *et al.*, 2015), thickness, and variable level of reserves. In the model, mobilizable leaf carbon is translocated during senescence (e.g. leaves becoming lighter).

##### Stem and woody biomass

Potential stem elongation is modelled using a linear function of day length multiplied by a Heaviside function for the effect of daily average air temperature (Powers *et al.*, 2006) (Eq. S5 at *JXB* online).

The woody growth potential is expressed in terms of total dry biomass production, which was computed from the average stem volume and specific stem dry weight, multiplied by the observed/simulated number of stems still alive. The stem volume depends on stem length and diameter/height ratio (*m*_DH_; Eq. S6 at *JXB* online) modified by a shape parameter *η*_St_ (Eq. S7 at *JXB* online), as stems are not exact cylinders.

##### Below-ground biomass

The stool and coarse and fine roots are the components of BGB modelled defining respective elongation rates, radial extension, and specific densities. Parameters of root extension and dry matter accumulation were calibrated against observed data (Cunniff *et al.*, 2015) on the basis of seasonal allocation (de Neergaard *et al.*, 2002) and respiration, as well as turnover (Rytter, 2001) rates of fine root dry matter.

#### Source formation 

##### Light interception

The genotype-specific light interception is described by a pseudo-3D architectural model (Cerasuolo *et al.*, 2013), which defines horizontal and vertical spatial distribution of leaves in a gap fraction model, characterizing LAI distribution by clumping (*Ω*) and profile shape (*η*) factors. The LAI is computed daily and the cumulative LAI is considered as *Ω*×*L*_c_(*z*), where *L*_c_(*z*) is the distribution of leaf area over the canopy depth (*z*). The light interception module describes the effect of canopy clumping on both direct and diffuse radiation (De Pury and Farquhar, 1997). The extinction coefficient for the diffuse radiation is calculated according to Goudriaan (1988), with weighted contributions from the three zones of a standard overcast sky. To simulate light interception, the canopy is divided into five layers, which are either uniformly or asymmetrically distributed. Within each layer, the ratio of sunlit/shaded leaf area is calculated to estimate the vertical variation of photosynthesis inside the canopy.

##### Photosynthesis and carbon pools

Photosynthesis is computed as the assimilation rate of CO_2_ using the maximum between an exponential function of the intercepted energy (APAR) and its potential absorption, modified by CO_2_ air concentration and air temperature (Goudriaan and van Laar, 1994). The effect of soil water availability on stomatal conductance and reduction in CO_2_ absorption is represented using a logistic function to describe the reduction of photosynthesis with decreasing relative soil water content (Sinclair, 1986).

Three different biochemical pools are simulated: first, a source pool of available carbohydrates (*C*_av_) composed of photosynthetic assimilates and remobilized reserves used for growth and maintenance processes; secondly, a source–sink pool of mobilizable carbohydrate reserves (e.g. starch) in leaves, wood, and stool; and finally, the sink pool of structural biomass, divided into AGB (stems, branches, and leaves) and BGB (stool, and coarse and fine roots).

#### Sink–source interaction 

##### Carbon allocation

The allocation of *C*_av_ is modelled as a combination of a sink–source balance and a hierarchical cascade [leaf>stem≈(pooled stool and coarse and fine roots)]. The respective sink strengths result from genetically determined growth potentials (see above) defined by the maximum rate of each organ’s dry matter accumulation and turnover (Genard *et al.*, 2008).

The total source (*C*_av_; Eq. 1a) to satisfy sink demands is calculated as the net daily integral of the difference between hourly leaf photosynthesis (*CH*_2_ *O*) and maintenance respiration of the respective tree organs (*R*_t_; Eq. 1b), plus the mobilizable reserves from leaves (*Lf*_Res_), woody biomass (*W*_Res_), and stool (*Stl*_Res_):

$$C_{\text{av}}=CH_{\text{2}}O-R_{\text{t}}+Lf_{\text{Res}}+W_{\text{Res}}+\alpha \times Stl_{\text{Res}}$$ (1a)

$$R_{\text{t}}=\left(m_{\text{Lf}}\times Lf+m_{\text{B}}\times B+m_{\text{Stl}}\times Stl\right)\times Q_{10}{}_{10}^{\frac{T_{\text{avr}}-T_{\text{Q10}}}{10}}$$ (1b)

A fraction of *Stl*_Res_ (*α*=0.04) can be mobilized for 20 d after budburst (Deckmyn *et al.*, 2008; ANAFORE Manual). If new assimilates exceed sink demands (Schapendonk *et al.*, 1998), the emerging surplus of assimilates is allocated to the reserve pools. The available carbohydrates for AGB (*AGB*_av_) are converted into leaf (*LfB*) and woody stem (*WSB*) biomass using their respective conversion factors (Penning de Vries *et al.*, 1983) and potential sink increases:

$$LfB\;=AGB_{av}\times \;LGrPt\;/\;ShGrPt,$$ (2a)

$$WSB\;=AGB_{av}\times \;SGrPt\;/\;ShGrPt.$$ (2b)

Here, *ShGrPt* represents the total shoot growth potential, and *LGrPt* and *SGrPt* the leaf and stem growth potentials, respectively.

*C*_av_ is partitioned between *AGB*_av_ and *BGB*_av_ using constant potential allocation coefficients, derived from the experimental evidence. These allocation coefficients change with stool size to account for increasing plant vigour during establishment and drought to increase root growth for better resource capture (Goudriaan and van Laar, 1994).

$$StlB=BGB_{av}\times StlGrPt/BGGrPt$$ (3a)

$$RtB=BGB_{av}\times \;RtGrPt/BGGrPt$$ (3b)

Stool and roots are assumed to turn over with different rates; stools are set to have a longevity, which corresponds to the stand/plant life-time (Bullard *et al.*, 2002b), while fine roots are set to a short mean residence time (0.25 years; Rytter, 2001).

Consecutively, *C*_av_ is allocated to the plant organs according to their respective sink strengths, defined by LAI and SLA, wood volume and density, stool mass, root growth, and turnover. Daily carbon allocation is, therefore, either limited by *C*_av_, or by the effective sink demand of assimilates (potential growth). At each time step, LUCASS balances the gain and consumption of carbon, estimates the conversion of *C*_av_ into growth, and calculates the produced biomass for each component (g m^–2^ upscaled to kg ha^–1^).

##### Effects of the environment

The soil hydrology is modelled using an energy balance approach combined with a two-layer soil water module (Richter *et al.*, 2006). The energy fluxes at the canopy surface are controlled by crop characteristics (LAI, stomatal resistance, canopy height), climatic variables, and soil hydraulic properties, e.g. water retention curves (see Supplementary Table S1 at *JXB* online), and resource capture (water uptake). The rooting depth is dynamic and is calculated using a constant crop-specific root advancement coefficient and maximum (plant×soil) rooting depth. The soil water balance, transpiration, and water uptake are calculated using the Penman–Monteith equation. The plant WS variable, *k*_WS_, is described by a non-linear, logistic function (Eq. S2c at *JXB* online) dependent on the relative water content between minimum and maximum plant-available soil water (Sinclair, 1986). Its curvature is determined by the WS parameter, *WSP* (Table 2), which was calibrated using the drought season (R1) data at ROTH. AGB/BGB partitioning is modified according to soil water availability (van Laar *et al.*, 1992).

**Table 2.** Alphabetical list according to process domain of model parameters used in LUCASS Symbols, definition, and units as well as source (reference, experimental evidence) are given.

Symbol	Definition	Units	Reference/comments
**Phenology**			
*C*_r_	Chilling requirement	d	Optimized
*d*_ac_	Number of day necessary to the crop to reallocate resources	d	Optimized
*dd*_*f*ill_	GDD for max stem filling rate	°C d	Calibrated
*dl*_0SER_	Stem elongation rate, intersect	d	Measured
*dl*_BtoBr_	Base photoperiod of buds becoming branches	d d^–1^	Assumed
*dl*_maxBtoBr_	Photoperiod for maximum rate of buds becoming branches	d d^–1^	Assumed
*NBuds*_0_	Initial bud number	–	Measured
*T*_B_	Base temperature for above-ground growth	°C	Perttu and Philippot (1996)
*T*_BG_	Base temperature for below-ground growth	°C	Assumed
*T*_bStE_	Base temperature for stem elongation	°C	Calibrated
*T*_c_	half-efficiency temperature	°C	Optimized
*T*_optBtoBr_	Optimum temperature for buds becoming branches	°C	Calibrated
**Morphology: sink formation**			
*a*_Stl_	Linear coefficient in the stool elongation rate	mm d^–1^	Calibrated
*a*_Stl/H_	Linear coefficient in the linear relationship of stem height–stool weight	m g^–1^	Calibrated
*b*_Rt_	Root elongation rate	m d^–1^ °C^–1^	Calibrated
*b*_Stl_	Constant coefficient in the stool elongation rate	mm d^–1^ °C^–1^	Calibrated
*b*_Stl/H_	Constant coefficient in the linear relationship of stem height–stool weight	m	Calibrated
*C*_BtoBr_	Maximum relative rate of buds producing branches	d^–1^	Calibrated
*c*_LER_	Leaf extension, constant	m	Porter *et al.* (1993)
*f*_*f*ill_	Power for stem filling rate	–	Calibrated
*ff*_maxBtoBr_	Maximum proportion of buds that produce new branches	–	Calibrated
*h*_0DH_	Relationship diameter/height intersect	mm	Measured
LAI_CShade_	Minimum LAI for shading to cause senescence	m^2^ m^–2^	Calibrated
*l*_distr_	Leaf layers distribution	–	Cerasuolo *et al.* (2013)
*L*_fShp_	Leaf shape factor	–	Measured
*L*_fWth_	Leaf width	m	Measured
*ls*_Br_	Relative reduction of branching with increased LAI	–	Calibrated
*m*_DH_	Relationship of diameter/height slope	mm m^–1^	Measured
*m*_LER_	Leaf elongation linear coefficient	m d^–1^	Porter *et al.* (1993)
*m*_SER_	Stem elongation rate, slope	m d^–1^	Measured
*NL*_Br_	Number of leaves per branch	–	Calibrated
*n*_Stlwt_	Power coefficient for the estimation of the stool weight factor	–	Calibrated
*n*_maxStlwt_	Stool weight at which the stool weight factor reaches its maximum effect	g m^–2^	Calibrated
*ρ*_AG_	Fraction of assimilates going to the above-ground organs	–	Measured
*ρ*_Rt_	Fraction of below-ground assimilates going to roots	–	Measured
*ρ*_St_	Specific stem weight	g m^–2^	Measured
*SLA*_max_	Maximum specific leaf area	m^2^ g^–1^	Measured
*SLA*_min_	Minimum specific leaf area	m^2^ g^–1^	Measured
*St*_max_	Max stem number given the initial number of buds	–	Calibrated
*η*_St_	Stems shape parameter	–	Assumed
*μ*_Br_	Porter mortality factor—lower asymptote	–	Porter *et al.* (1993)
*μ*_W_	Branches and stems aging death rate	d^–1^	Measured
*μ*_WRes_	Percentage of woody reserves lost during the harvest	g g^–1^	Calibrated
*σ*_Rt_	Root dry matter per unit length	g m^–1^	Calibrated
*σ*_Stl_	Stool structural dry matter per unit length	g m^–1^	Calibrated
*W*_*l*oss_	Percentage of dry matter lost during the harvest	–	Calibrated
**Physiology: source formation**			
Light interception			
*α*	ELADP quadratic coefficient	–	Observed
*β*	ELADP linear coefficient	–	Observed
*γ*	ELADP constant	–	Observed
*η*	Shape parameter for the vertical leaf area distribution	–	Cerasuolo *et al.* (2013)
*Ω*	Clumping index	–	Cerasuolo *et al.* (2013)
*μ*_T_	Temperature-driven increase of senescence	d^–1^	
*μ*_maxShade_	Maximum shading-induced senescence rate	d^–1^	Calibrated
*μ*_Shade_	Shading-induced increase of senescence rate per unit of LAI	d^–1^	Calibrated
*μ*_WS_	Water stress-driven increase of senescence	d^–1^	Calibrated
Assimilation and respiration			
*A*_max_	CO_2_ potential assimilation rate at light saturation	g (CO_2_) m^–2^ s^–1^	Bonneau (2004)
*P*_cmax_	Max photosynthetic rate capacity	μg (CO_2_) m^–2^ s^–1^	Bonneau (2004)
*p*_Lf_	Percentage of single leaves produced by new flushing buds	–	Calibrated
*Q*_10_	Responsiveness of respiration at a temperature of 10 °C	–	Sampson and Ceulemans (2000)
*r*_b_	Boundary layer resistance	s m^–1^	Calibrated
*R*_BG_	Maintenance respiration rate of roots	g (glucose) d^–1^	Vivin *et al.* (2002)
*R*_D_	Dark respiration	μg (CO_2_) m^–2^ s^–1^	Kaipiainen (2009)
*Res*_max_	Maximum reserve fraction	–	Calibrated
*Res*_maxStl_	Maximum reserve fraction of stool dry matter	–	Calibrated
*R*_Lf_	Maintenance respiration rate of leaves	g (glucose) d^–1^	Vivin *et al.* (2002)
*r*_s,min_	Minimum stomatal resistance	s m^–1^	Bonneau (2004)
*R*_St_	Maintenance respiration rate of stems	g (glucose) d^–1^	Vivin *et al.* (2002)
*T*_bC_	Base temperature in CO_2_ assimilation	°C	Assumed
*T*_maxC_	Maximum temperature in CO_2_ assimilation	°C	van Laar *et al.* (1992)
*T*_minC_	Minimum temperature in CO_2_ assimilation	°C	
*T*_optC_	Optimal temperature in CO_2_ assimilation	°C	
*WSP*	Water stress parameter	–	Calibrated
*Γ*	CO_2_ compensation point at 25 °C	μmol mol^–1^	Xu *et al.* (2008)
*ξ*_GW_	Conversion of assimilates to biomass	g (glucose) g^–1^	Penning de Vries *et al.* (1983)
*φ*_pot_	Quantum efficiency of photosynthesis	μg CO_2_ J^–1^	Bonneau (2004)
*σ*	Scattering coefficient of leaves for PAR	–	Goudriaan (1988)

The effects of WS on leaf emergence and elongation rates, and stem and leaf mortality are also considered as a function of *k*_WS_ and respective potential rates. Buds and branches follow the same dynamics as leaves, but the mortality rate of branches is assumed to be 10 times lower than that of leaves. The mortality rate of stems is also computed as the sum of natural turnover and death rate caused by water and shading stress; however, stem mortality is less than that of leaves (*μ*_WS/T_ 2×10^–4^ and 1.2×10^–3^ d^–1^, respectively).

### Calibration and parameter ranking

The model inputs divide into environmental variables and process parameters: (i) field location (longitude, latitude, etc.) and soil characteristics; (ii) management data (irrigation, harvest days, number of years per growth cycle); (iii) (hourly) weather data, e.g. solar radiation, mean air temperature, wind speed and direction, rainfall and air humidity; and (iv) genotype-specific growth parameters.

#### Model calibration 

The parameters of the growth model were calibrated using genotype-specific experimental data where values from the literature were not available (Table 2). Process-specific evidence was used to calibrate development and morphology either through direct measurements (e.g. leaf emergence and senescence) or through parameter estimation involving model data fitting (e.g. budburst, stem height, and stem diameter). Model cross-validation was performed using a time series of the variables not used for calibration (e.g. canopy height, stem biomass). The photosynthesis parameters (Bonneau, 2004) were calibrated to match total biomass production and turnover.

Parameters of the budburst model (Eq. S1 at *JXB* online) were calibrated using ROTH data from the first rotation cycle (R1, 2010–2011), while parameters for stem height/diameter relationships were estimated using data from both locations (ABER and ROTH, 2010–2011). LUCASS (remaining parameters) was calibrated for potential productivity using data from ABER assuming that water was unlikely to limit growth and carbon partitioning, especially in R2. The flux parameters for carbon allocation were calibrated using morphological components of AGB (leaf and stem weight) and BGB (stool and fine root weight). In a final step, the WS effect on biomass production was calibrated using a time series of data collected at ROTH in R1 (e.g. LAI).

#### Sensitivity analysis 

A global SA was performed for all varieties and both sites for potential (no water stress, NWS) and actual (WS) growth, and the model response was determined for the first and second coppice rotations (R1 and R2). The aim was to understand which growth parameters had a significant impact on final yield, and whether it changed with the environment, age of stand or phenotype. All of the 78 parameters (Table 2) were varied in a one-at-a-time modus using the Morris method (Morris, 1991). Assuming that all parameters were normally distributed (Richter *et al.*, 2010), the window of their variation was set to a respective standard deviation of 10%. The estimated average response strength (*μ*) for each parameter represents its overall effect on the model outcome (e.g. final yield). Its standard deviation (*σ*) represents the response spread estimating higher-order effects (non-linearity, parameter interactions). Both *μ* and *σ* were calculated over six different trajectories (individual one-parameter-at-a-time simulations) and using six levels (granularity of the explored parameter space) (Richter *et al.*, 2010).

### Data analysis and model validation

Data were analysed with Genstat^TM^ 14 (Payne *et al.*, 2011) to examine the influence of location and varieties using a two-way ANOVA. Linear regressions were performed using Sigmaplot (version 12.0, 2011). The model was validated against yield data from the second growth cycle at ROTH, and independent datasets for three of the four varieties at LARS and ROTH. The goodness of simulations to match experimental data for the two dedicated trials was characterized with the residual mean square error (RMSE). The coefficient of determination, the model efficiency (ME), RMSE, bias [mean difference (MD)] and *r* ^2^ were calculated according to Smith *et al.* (1997).

## Results

All results fell into a distinct pattern due to significantly different climatic conditions during the two rotations where R1 was distinctly drier than R2, which translated into high WS, especially in 2010, and low WS, especially in 2012. These conditions were exacerbated by site differences and reached almost potential NWS conditions in ABER during 2012, while ROTH had strong WS conditions during R1 growth.

### Sensitivity Analysis

#### Identification and ranking of key parameters

**Fig. 2.**
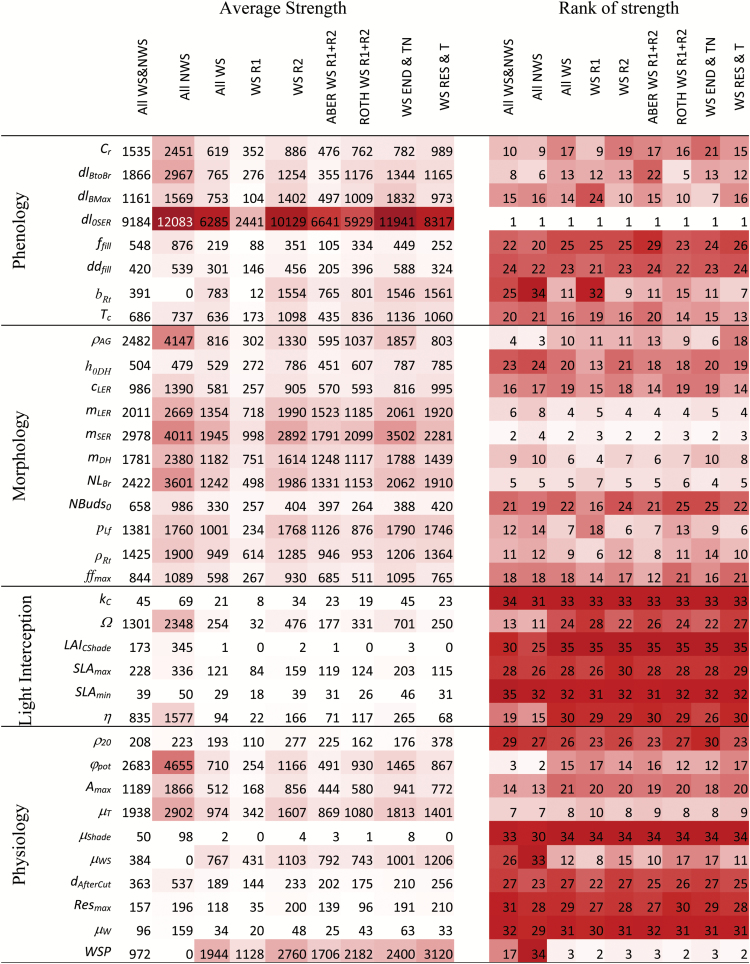
Heat map for the average of response strength (*μ*) estimated using the Morris method and ranking calculated for all varieties together or separated according to potential and water-limited conditions (all WS and NWS, all NWS, and all WS). Average sensitivity was calculated under water-limited conditions for the first (WS R1) and second (WS R2) rotations separately, for sites considering both rotations (ABER WS R1+R2 and ROTH WS R1+R2), and across similar canopy phenotypes (END & TN, Endurance and Terra Nova; RES & T, Resolution and Tora). Colour intensity increases with increasing response strength but is lower for higher ranks.

**Fig. 3.**
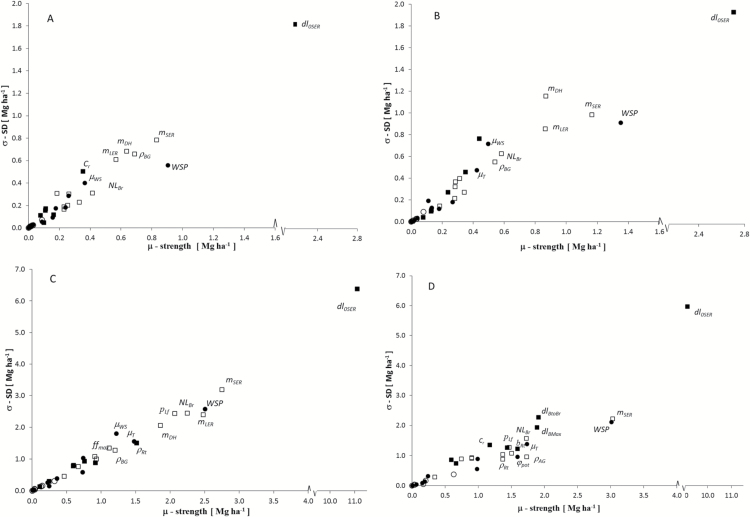
Morris sensitivity measures (μ*, *σ*) under water-limited production to random changes of 34 model parameters averaged across all genotypes for ROTH (A, C) and ABER (B, D) during the first and second rotations, respectively. Symbols represent pheno logical (closed squares) and morphological (open squares) sink-related parameters, and physiological (closed circles) and other source-related parameters (open circles).

The heat map details (see Supplementary Fig. S3 at *JXB* online) showed a clear pattern of high sensitivity under potential (NWS) and low WS (R2) growing conditions, which translated clearly into the aggregated averages (Fig. 2). Considering a model response threshold of about 1000kg ha^–1^ per 10% parameter change (4–5% yield potential), the SA revealed that yields were affected by up to 20 parameters (under NWS). Under conditions of growth-limiting WS, the number of parameters with significant effects on yield dropped to fewer than 10. However, these ranked consistently high when considering the average response to their variation across sites (ABER, ROTH), age (R1, R2), and canopy phenotype. Model sensitivity was higher in the second (R2) than in the first (R1) rotation (Fig. 3), most likely due to lower WS. Differences between sites were small overall and affected only a few parameters [day length associated with buds turning into branches (*dl*_BtoBr_); AGB/BGB partitioning (*ρ*_AB_), and quantum efficiency (*φ*_pot_)]. Most of these parameters reflected experimental evidence and measurable crop traits defining sinks and sources.

#### Sink formation: phenomorphology

The onset of stem elongation (*dl*_0SER_) was identified as the overall most important yield determining (phenological) parameters at both sites and for all conditions. It was followed by closely related morphological sink determinants, such as stem elongation rate (*m*_SER_) and diameter/height coefficient (*m*_DH_). The fraction of total biomass allocated to AGB (*ρ*_AG_) also ranked among the strongest effects, emphasizing the importance of AGB/BGB partitioning. The fraction allocated to roots (*ρ*_Rt_) had an equally large effect (0.7–2.3 Mg ha^–1^). These sink parameters had the most stable ranking across most of the subsets of the SA; exceptions were rank changes for *ρ*_AG_ with site and phenotype.

Phenological parameters that determine budburst [chilling requirement (*C*_r_); base temperature (*T*_c_)] showed a very inconsistent and contrasting behaviour. *C*_r_ ranked higher overall for potential than water-limited growth, which was also reflected in its higher rank in the wet second rotation. Differences between sites were marginal, but both parameters were slightly more important for ABER than for ROTH (see Supplementary Table S4 at *JXB* online); however, *C*_r_ ranked on average slightly higher for the open canopy phenotype (Fig. 2).

#### Source formation

Source-related parameters (light interception, photosynthesis) were on average less sensitive than sink-related parameters. Parameters of canopy structure (Ω*; η*) were identified as important under potential (2.3 and 1.6 Mg ha^–1^) but not under water-limited production (<0.5 Mg ha^–1^). On the other hand, parameters determining light interception, e.g. the number of leaves and leaf elongation rate (>2 Mg ha^–1^) ranked consistently high, irrespective of the site, rotation, or phenotype. The sensitivity of photosynthesis, quantum efficiency (*φ*_pot_), was on average more than twice that of the CO_2_ potential assimilation rate at light saturation (*A*_max_), emphasizing light conversion at low light levels to be crucial for willow production in the UK. There was a difference between sites (see below) and phenotypes; the change of *φ*_pot_ was more important in large, closed-canopy phenotypes (END and TN).

#### Environmental effects

The overall parameter effects on yield were only marginally higher at ROTH than at ABER (0.73 and 0.81 Mg ha^–1^; Fig. 3), but model sensitivity was higher in R2 than in R1, which reflected the wetter growth conditions in R2, while R1 was characterized by WS aggravated by higher cumulative radiation (Table 1). The relative sensitivity to changes of physiological parameters (photosynthesis) was similar for both sites when tested for potential production (NWS; Supplementary Table S4 at *JXB* online). The effects of WS reduced the overall sensitivity to changes of other physiological parameters (light interception and photosynthesis).

The SA revealed interactions between process and site, e.g. resulting in different parameter rankings related to temperature [budburst and senescence (*μ*_T_)] and water, both marginally more important at ROTH than at ABER. The high ranking of *WSP* did not translate into similar differences caused by variation of WS-induced senescence (*μ*_WS_) (Fig. 2). At ROTH, the average effect of source-related parameters on yield, such as light interception (onset of branching, *dl*_BtoBr_, number of leaves per branch, *NL*_Br_, Ω, and *η*) and photosynthesis (*φ* and *A*_max_) ranked lower than at ABER, which could reflect an interaction of light (lower radiation) and water availability. In contrast, parameters related to carbon allocation (sink size) ranked higher at ROTH than at ABER.

### Model calibration and cross-validation

#### Phenology, light interception, and LUE

Budburst parameter values showed a small variation among varieties (see Supplementary Table S3 at *JXB* online) with an average value of 6.1±0.5 °C and –18.1±0.4 for *T*_C_ and *C*_r_, respectively. The model explained an overall 79% of the variance in budburst date at ABER. In 2013, budburst showed a reduced goodness of fit by more than 10% at both sites as temperatures were outside the range of calibration.

**Fig. 4.**
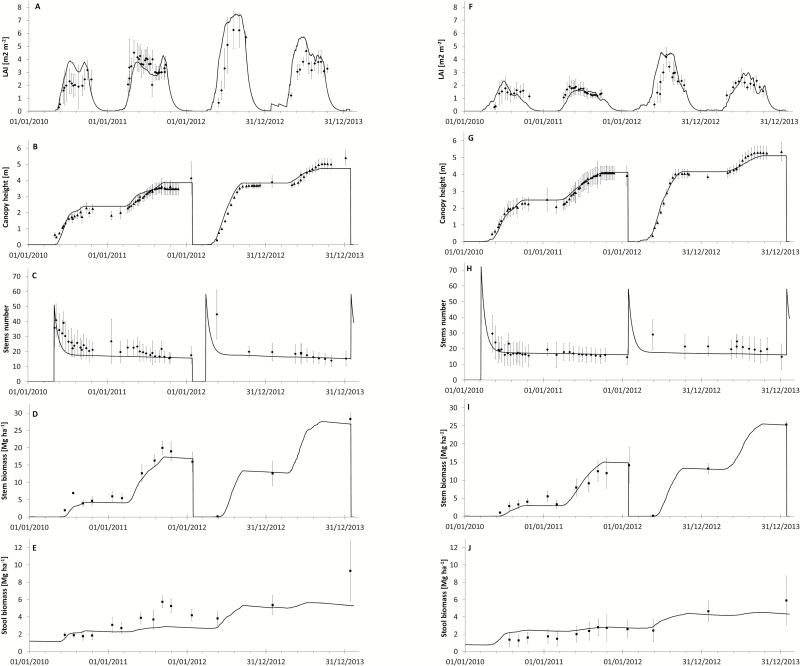
Observed (filled symbols) and simulated (solid line) LAI, canopy height, stem number, and accumulated stem (AGB) and stool (BGB) yield of Endurance (A–E) and Tora (F–J) grown at ROTH over two consecutive rotations (2010–2011 and 2012–2013). The error bars represent the standard deviations of the experimental values (*n*=4).

Light interception is the result of a complex process of leaf area formation (Eq. S2–S4 at *JXB* online). Leaf area was first calibrated at ABER using only destructive LAI measurements, and then recalibrated for WS against the experimental evidence of LAI at ROTH during the first rotation (Fig. 4A, F).

The LAI simulation at ROTH (Endurance and Tora in Fig. 4A, F and Resolution and Terra Nova in Supplementary Fig. S4 at *JXB* online) was better during the first rotation than during the second (respective RMSE values for Endurance were 0.95 and 1.78, Table 3). This was mainly due to delayed canopy development after coppicing in January 2012. Overall, the model reflected the genotypic differences between canopy types quite well, but described LAI better for non-coppiced than for coppiced years (RMSE values of 0.76 and 1.26, respectively).

**Table 3.** Goodness of fit for modelling growth indicators LAI, canopy height (*h*_c_), number of stems (*n*_stems_), biomass of stem (*B*_stem_) and stool (*B*_stool_), and overall yield of the four willow varieties grown at ROTH for the first (R1, 2010–2011) and second (R2, 2012–2013) rotation were used for validation. RMSE, residual mean square error; MD, mean difference; ME, modelling efficiency; *R* ^2^, certainty.

Variety	Indicator	RMSE		MD (O–S)		ME		R^2^	
		R1	R2	R1	R2	R1	R2	R1	R2
Endurance	LAI (m^2^ m^–2^)	0.95	1.78	–0.09	–1.24	0.07	–0.45	0.25	0.38
	*h*_c_ (m)	0.30	0.26	–0.18	–0.08	0.89	0.96	0.95	0.97
	*n*_stems_ (m^–2^)	6.39	7.24	4.40	2.97	0.02	0.15	0.55	0.95
	*B*_stem_ (Mg ha^–1^)	1.98	0.87	1.49	0.39	0.90	0.99	0.96	1.00
	*B*_stool_ (Mg ha^–1^)	1.37	2.38	0.85	1.74	–0.03	–0.06	0.62	0.58
Resolution	LAI (m^2^ m^–2^)	0.70	0.93	0.45	–0.40	–2.67	–0.23	0.03	0.37
	*h*_c_ (m)	0.29	0.32	0.16	–0.18	0.94	0.94	0.96	0.96
	*n*_stems_ (m^–2^)	6.64	4.04	3.92	2.04	–0.18	–0.15	0.78	0.66
	*B*_stem_ (Mg ha^–1^)	1.86	1.20	0.39	–0.91	0.89	0.99	0.90	1.00
	*B*_stool_ (Mg ha^–1^)	0.80	1.57	0.53	1.19	–0.10	–0.01	0.84	0.95
Terra Nova	LAI (m^2^ m^–2^)	0.79	1.17	0.35	–0.63	–0.96	–1.07	0.03	0.35
	*h*_c_ (m)	0.23	0.31	0.14	0.16	0.94	0.93	0.96	0.96
	*n*_stems_ (m^–2^)	3.87	2.58	–0.19	1.18	0.30	0.11	0.74	0.46
	*B*_stem_ (Mg ha^–1^)	1.73	0.99	–0.36	0.22	0.91	0.99	0.95	0.99
	*B*_stool_ (Mg ha^–1^)	1.02	1.30	–0.74	–1.29	–0.02	–0.06	0.94	1.00
Tora	LAI (m^2^ m^–2^)	0.45	1.17	0.10	–0.61	–0.63	–1.54	0.15	0.28
	*h*_c_ (m)	0.16	0.21	–0.03	0.03	0.97	0.98	0.98	0.98
	*n*_stems_ (m^–2^)	2.28	5.15	0.43	4.21	0.44	–1.45	0.76	0.76
	*B*_stem_ (Mg ha^–1^)	1.69	0.11	0.04	0.11	0.85	1.00	0.95	1.00
	*B*_stool_ (Mg ha^–1^)	0.64	0.96	–0.52	0.49	–0.33	0.56	0.91	0.91
All*	*B*_stem_ (Mg ha^–1^)	1.81	0.89	0.38	–0.05	0.90	0.99	0.92	0.99
	*B*_stool_ (Mg ha^–1^)	1.00	1.64	0.06	0.53	0.22	0.28	0.26	0.41

*Due to a small number of observations during R2 for biomass (*n*=3 compared with >10 for the other indicators), the data were pooled together to give an overall estimation.

**Fig 5.**
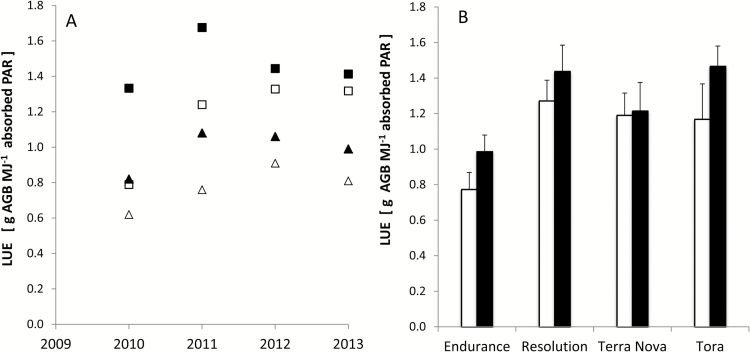
Simulated average light use efficiency (LUE, g AGB MJ^–1^ APAR) during the time course of the experiment (2010–2013) for the varieties Tora (squares) and Endurance (triangles) at ROTH (open symbols) and ABER (closed symbols) (A) and averaged for all varieties at both sites (B). The error bars represent the standard deviation.

The parameters for photosynthesis were estimated against total biomass (e.g. Fig. 4D, E, fine roots), and *A*_max_ in the range of 18.9–23.3 μmol m^2^ s^–1^ matched the sink demand well. For Terra Nova, we calibrated a value similar to Endurance as both had a similar canopy. For comparison, photosynthesis was also expressed in terms of LUE, based on annual woody AGB (stem yield) and simulated intercepted PAR (Fig. 5A) and averaged over all years (Fig. 5B). LUE was 6% lower during R1 compared with R2 at ROTH but not at ABER. LUE actually showed a site×year×phenotype effect. While LUE for narrow-leaved Tora recovered during 2011, it remained low for broad-leaved Endurance at ROTH due to drought. This was reflected in the respective yield drops in comparison with the R2 validation data (see below, 13 and 28%; see Fig. 7). At ABER, there were similar LUE increases for both phenotypes during 2011, which compensated for the overall low initial efficiency (2010), although not so at ROTH.

**Fig. 6.**
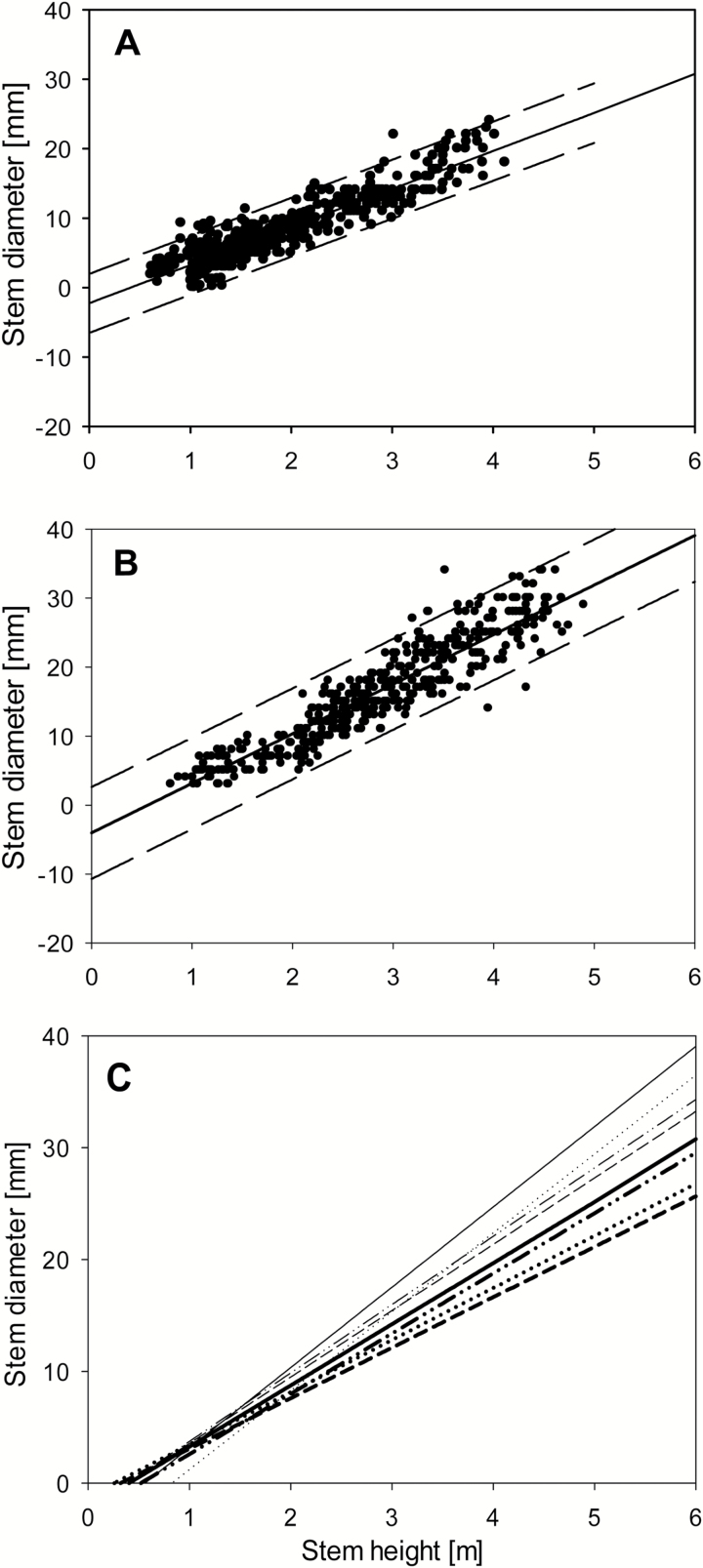
Correlations between stem diameter and stem height for Endurance at ROTH (A) and ABER (B), and sketched for all willow varieties (Endurance **––**, Resolution - - -, Terra Nova –··–, Tora ······) for ROTH (bold lines) and ABER (fine lines) (C).

**Fig. 7.**
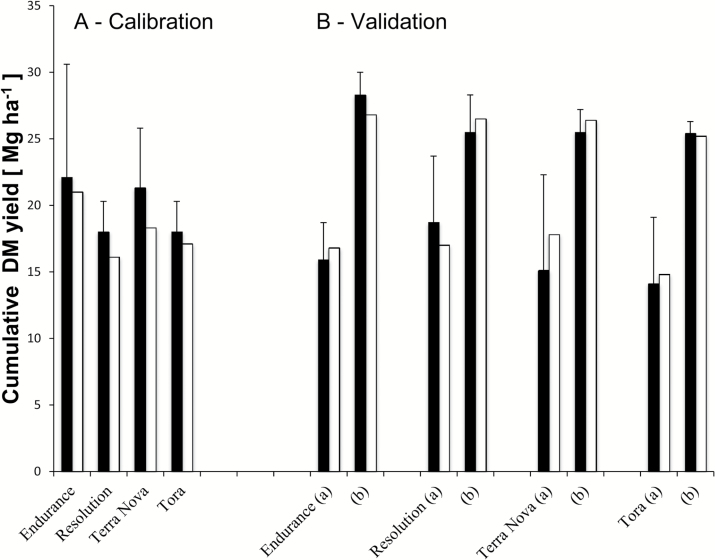
Observed (filled columns) and simulated (open columns) accumulated yields of broad-leaved (Endurance, Terra Nova) and narrow-leaved (Resolution, Tora) willow varieties for calibration during the first coppice cycle (R1; 2010–2011) at ABER (a) and ROTH (b) (A) and validation over the second coppice cycle (R2; 2012–2013) at ROTH (B). DM, dry matter. The error bars represent the standard deviation of the observed yields.

#### Sink formation: carbon allocation

Potential stem elongation rates were initially calibrated using stem height data during 2012 at ABER to avoid bias due to carbohydrate limitations and WS. The parameter values for stem elongation (Eq. S5 at *JXB* online) were then optimized through iteration using ABER genotype-specific growth data (time series) during the first rotation (2010–2011). These parameters fitted well with the heights observed at ROTH (Table 3). The agreement between computed and observed stem extension rates was reflected in the stem growth dynamics at ROTH, in particular for the variety Tora (Fig. 4G).

Stem number was strongly affected by the environment, with numbers significantly smaller at ABER than at ROTH (*P*<0.01) during 2010–2011, and by genotype (*P*<0.001) with Endurance having the most and Resolution the fewest. A significant interaction between sites and varieties (*P*<0.01) was related to greater range in stem numbers among varieties at ROTH compared with ABER.

The relationship between stem height and diameter also showed a strong interaction between sites and varieties (Fig. 6; *P*<0.001). The data showed two separate groups, one for ROTH where stems were thinner and the other for ABER where stems were thicker, for an equivalent height. The parameters *m*_DH_ and *c*_DH_ were evaluated for each variety at each site from the first rotation data (see Supplementary Table S5 at *JXB* online). These observations suggested a combination of site-specific parameter values for AGB/BGB partitioning and initial stem numbers and height/thickness (*m*_DH_).

Destructive harvest data from both sites (R1) were used to approximate the partitioning between AGB and BGB (including coarse but not all fine roots) in all varieties (e.g. Fig. 4D, E, I, J; see also Supplementary Fig. S4N, O, S, T at *JXB* online). The four varieties allocated between 80% (Endurance) and 90% (Resolution) of the dry matter to the AGB. Stem and stool biomass data at the end of rotations showed that all varieties allocated a smaller fraction of assimilates to BGB during R2 compared with R1. For Endurance, this dropped from 20 to 15%, while Tora reduced allocation from 15.4 to 11.3%.

### Model validation

#### Validation using the variety trial at ROTH

The model validation was first done by comparing the average LAI, stem height and number, and AGB/ BGB yields measured in the second rotation of growth (R2) at the dedicated variety trial at ROTH with the corresponding simulations (Table 3). The model predicted the differences in LAI between varieties well, with values for Endurance being highest and for Tora the lowest (Fig. 4). However, LAI modelling efficiency was low due to a phase shift of regrowth during the first year of the second rotation. Separating coppicing from non-coppicing years improved the statistical indicators of the prediction of LAI data (RMSE=0.76; MD=–0.23; *R* ^2^=0.61).

A management×site effect was observed in terms of different stem numbers and height/diameter ratio between ABER versus ROTH across all four varieties (Fig. 6). These observations suggested site-specific parameter values for AGB/BGB partitioning and initial stem numbers and height/thickness (*m*_DH_).

The final yield predictions agreed in a satisfactory way with the empirical data at ROTH for all four willow varieties for both rotations (R1, cross-validation, and R2, validation) (Fig. 7). This result was consistent with the fact that for all varieties we observed a high ME and *R* ^2^ (>0.85, Table 3) for stem biomass. The daily simulations for LAI, stem height, and biomass, as well as stem number, were within the 95% confidence interval of mean observations of these variables (Fig. 4). The model was able to catch the behaviour of the studied traits throughout the growing seasons for all studied willow varieties. The comparison between measured and simulated BGB was satisfactory for most varieties (Fig. 4E, J). ME was high for canopy height (all >0.9) and stem biomass yield (overall due to small number of samples ~0.99), and acceptable for BGB (overall ~0.28), while it was low for LAI and stem number (Table 3), due to slight asynchronies (Fig. 4).

It is interesting to observe that in wet years (2012; see Supplementary Table S2 at *JXB* online) broad-leaved varieties (e.g. Endurance, Fig. 4D) performed better than the others (e.g. Tora, Fig. 4I) but were more sensitive to WS in 2010. This is very clearly summarized in the yield calibration and validation (Fig. 7): in R1, low yields (7–11 Mg ha^–1^ year^–1^) reflected an overall establishment but also a drought (ROTH vs ABER) effect, mainly for broad-leaved varieties (END and TN, 29%). In comparison with R1 yields, R2 showed high yields at ROTH (12–14 Mg ha^–1^ year^–1^) with no drought effect, and establishment gains were larger for narrow- than for broad-leaved varieties (44 vs 24%). The narrow-leaved variety Resolution performed well in both rotations, irrespective of WS (see Supplementary Fig. S4 at *JXB* online), displaying an overall interesting G×E interaction. During the second rotation, final yields in all varieties were significantly lower at ABER than at ROTH. However, ABER second-rotation data could not be used for validation as the crop suffered exceptional wind damage that the model could not account for. Significant differences between genotypes (*P*<0.001) established Endurance as the best yielding variety in both locations, in spite of its susceptibility to drought.

#### Validation with further yield data

**Fig. 8.**
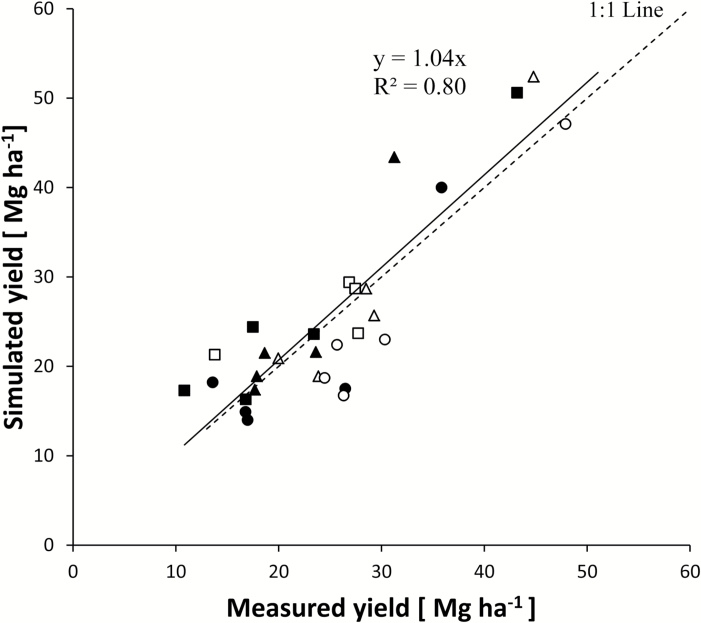
Correlation between measured and simulated 2- and 3-year coppice biomass yields for the three willow varieties from trials at ROTH (closed symbols) and LARS (open symbols). Endurance, squares; Resolution, triangles; Tora, circles.

Simulated and measured final biomass yields for the three varieties compared well at ROTH and LARS, irrespective of the length of the coppicing cycle (Fig. 8). The overall correlation between measured and simulated biomass yields across both locations was good (*R* ^2^=0.80) and the average difference was small and the ME high (MD=0.68 Mg ha^–1^; ME=0.7). Most of the predictions were concentrated near the 1:1 line, proving that the model was able to reproduce actual yields. It showed a slight bias towards lower yields at LARS and overestimated yields at ROTH.

## Discussion

The process-based model LUCASS characterized phenotypic carbon sinks and implemented a sink–source interaction to describe yield formation for different SRC willow genotypes. The novelty of this approach lies in its simplicity to parameterize the size of various sinks using phenotype-specific morphological characteristics. Calibrated and validated with data from sites across the UK, the model was able to illustrate the underlying system behaviour in terms of source and sink dynamics, and predicted final yields reflecting genotypic and environmental differences.

### Key productivity parameters

Compared with other models (Deckmyn *et al.*, 2008; Amichev *et al.*, 2011; Tallis *et al.*, 2013), LUCASS has fewer parameters, and fewer than 20 proved to be crucial for yield formation (Fig. 2). The SA identified the onset of stem elongation, stem elongation rate, and diameter as key parameters for yield formation and indicators of vigour (Kauter *et al.*, 2003; Volk *et al.*, 2006). Parameters of early development, e.g. chilling and forcing functions (Cesaraccio *et al.*, 2004; Fu *et al.*, 2013), were confirmed to be important at sites with a mild winter climate (ABER and LARS). Despite the importance of the start of spring growth (Cannell and Smith, 1983; Weih, 2009), it is not entirely clear whether chilling has a physiological role (Horvath *et al.*, 2003; Rohde and Bhalerao, 2007) in addition to cold hardiness (Morin *et al.*, 2007). Ongoing investigations will elucidate whether chilling should be modelled for temperate tree species (Fu *et al.*, 2012). The role of photoperiod (Fu *et al.*, 2012) needs testing over a range of latitudes, as the sites studied here were similar. Nevertheless, this study did show that photoperiod was important for canopy development in terms of branching (*dl*_BtoBr_) and stem elongation (*dl*_0SER_).

This analysis also showed that early budburst does not necessarily mean faster canopy development. Despite initial delays after coppicing, e.g. 2012, modelled and observed LAIs reached their maxima at similar times and values, and the disparity had no impact on biomass production. A late spring start was apparently compensated for by faster leaf growth when environmental conditions became favourable (Weih, 2009). Thus, although budburst date is important for modelling willow development (Savage and Cavender-Bares, 2011), it remains debatable whether its accuracy is also important for predicting yield (Tallis *et al.*, 2013).

### Genotypic differences for routes to high yields?

#### Source formation

The variation of willow yield proved highly sensitive to parameters of LAI distribution and genotypic canopy characteristics, confirming that stem dynamics and biomass yield are strongly influenced by radiation distribution within the canopy (Ceulemans *et al.*, 1996; Bullard *et al.*, 2002a; Tharakan *et al.*, 2008; Cerasuolo *et al.*, 2013). Photosynthesis parameters (*A*_max_, *φ*_pot_) differed across genotypes (Bonneau, 2004; Andralojc *et al.*, 2014) and were also confirmed as an important source of yield variation (Figs 2 and 3). Quantum efficiency (*φ*_pot_) consistently caused a larger model response than *A*_max_; however, both seemed to be strongly related, as found by Andralojc *et al.* (2014) and Kaipiainen (2009). Genotype ranking, according to photosynthetic capacity at the plant level, was dominated by leaf area, but genotypes realized similar biomass with different strategies, either through high photosynthetic rate or large leaf area, confirming previous results (Andralojc *et al.*, 2014).

LUE is a key physiological indicator, usually expressed in terms of woody AGB per unit absorbed PAR, which ranged from 0.6 to 1.7g m^–2^ MJ^–1^ (Fig. 5A). Mean LUE was significantly higher at ABER (Fig. 5B), indicating its interaction with drought and senescence (Savage *et al.*, 2009), canopy duration, and leaf abscission (Weih, 2009), which can affect cumulative photosynthesis (Philippot, 1996). These site-specific differences due to WS varied between broad- and narrow-leaved varieties (Endurance and Tora, respectively). Tora evaded drought by means of a smaller leaf number (Cerasuolo *et al.*, 2013) and LAI (Fig. 4F). The lowest LUE values were calculated for regrowth after first coppicing (2010), which was aggravated by drought at ROTH, especially for Endurance with its large canopy (–41%). *In situ* measurements under a controlled water supply showed a similar drop in photosynthetic efficiency (–33 to –60%; (Bonneau, 2004)). The range of average LUE (0.77–1.47g MJ^–1^), which was significantly different between sites (*P*<0.01) and varieties (*P*<0.001), agreed with the range of simulated values (Jing *et al.*, 2012) and other estimates (Bullard *et al.*, 2002a; Sannervik *et al.*, 2006; Tallis *et al.*, 2013). A large canopy increased a variety’s sensitivity to WS, e.g. the lowest LUE of Endurance irrespective of location, but achieved high yields in wet conditions. The effects of senescence on nutrient remobilization (Fracheboud *et al.*, 2009) and dry matter loss through respiration (de Neergaard *et al.*, 2002) can complicate the G×E interaction under variable climate conditions.

#### Sink formation

Morphological characteristics were the most important to identify high-yielding genotypes especially under WS conditions. The high sensitivity of these sink parameters across both sites (Figs 2 and 3) confirmed their importance for yield formation (Larsson, 1998). Traits such as stem number, height, and diameter, as well as leaf size and form, are also easily measured in high-throughput screenings (Sennerby-Forsse and Zsuffa, 1995; Bullard *et al.*, 2002b; Martin and Stephens, 2006; Sannervik *et al.*, 2006; Verwijst *et al.*, 2012).

Our results confirmed earlier findings that Endurance has the thickest stems while Resolution has the thinnest (Cunniff *et al.*, 2011). The genotype-specific relationship between stem diameter and height (Fig. 6) is an excellent example of integrating plant characteristics and environment influence (G×E interaction). Stem diameter increases with the length of the coppicing cycle and is an important determinant for harvestable wood volume (Bullard *et al.*, 2002b; Amichev *et al.*, 2011).

Moreover, parameter values for the potential allocated AGB were considered to be ~10–12% lower than those estimated from destructive measurements. This was due to the concurrence of two factors: around 30–40% of the net primary production produced by basket willow was used below ground, in particular on fine roots due to their high turnover rate (Rytter, 2001). Experimental evidence provided by soil cores collected at ROTH showed that actual fine-root biomass was up to three times that from destructive samples, e.g. Endurance 873 vs 283g m^–3^, respectively (Cunniff *et al.*, 2015). These root cores also showed that willows had a 65% greater fine-root volume when grown at ABER compared with growth at ROTH.

The analysis of the experiments showed differences in carbon storage in BGB (Cunniff *et al.*, 2015), which could have affected the regrowth dynamics (Sennerby-Forsse and Zsuffa, 1995; Tharakan *et al.*, 2008; Verwijst *et al.*, 2012). Poor yields of Tora under drought were shown to be concurrent with low initial BGB. Root biomass could be a key trait to mitigate such circumstances (Ceulemans *et al.*, 1996). Tora showed great resilience to high yield in the second growth cycle by building up a comparable fine-root mass (Cunniff *et al.*, 2015). However, the G×E interaction was not conclusive: in spite of more investment of carbon in BGB at ABER, the vigour after coppicing in 2012 dropped considerably (Cunniff *et al.*, 2015). Experimental data also showed significant differences in biomass allocation among varieties (*P*<0.001) and in the interaction between site and variety (*P*<0.05) (Cunniff *et al.*, 2011, 2015). Stem numbers were possibly related to soluble sugars/starch availability, but were difficult to separate from management effects (cut-back) at ABER, which resulted in a smaller stool volume with fewer buds to develop into new stems (Cunniff *et al.*, 2014).

Further analyses of the underlying physiological processes are needed to justify different modelling approaches for early development (Fu *et al.*, 2012) and the impact on early growth (Tharakan *et al.*, 2008; Verwijst *et al.*, 2012) and yield formation. A systematic budburst delay after coppicing can be expected (Verwijst *et al.*, 2012). Bud and stem numbers could be influenced by stool size (management effect), as well as starch and sugar contents (Tschaplinski and Blake, 1995; Cunniff *et al.*, 2014). This and evidence in regard to regrowth after coppicing (Tschaplinski and Blake, 1994; Von Fircks and Sennerby-Forsse, 1998) suggest model expansion to describe the number of buds bursting as a function of readily available carbohydrate.

BGB characterization within the system is essential (Karp and Shield, 2008), but few data exist for roots of SRC (Rytter, 2001; Pacaldo *et al.*, 2013; Agostini *et al.*, 2015; Cunniff *et al.*, 2015). Destructive harvests represented only part of the below-ground allocation underestimating fine root biomass between 16 and 24% (Cunniff *et al.*, 2015). Biomass in the fine root mass in a 1 m profile ranged from 3.56 to 6.46 Mg ha^–1^ for Tora and Endurance, respectively (unpublished data), a fraction that is likely to turn over fast (Rytter, 2001).

#### Source–sink interactions under different environments

Within the sink–source interaction, LAI and stem growth play the key roles for potential production, balancing the available carbohydrate for resource capture and harvestable biomass. A hierarchy of dry matter allocation to leaves over stems was needed to enable sufficient light capture. In the model, LAI is influenced by budburst and base temperature for leaf growth, which usually precede the day length threshold for stem elongation. Sufficient allocation of carbohydrate to leaves (and fine roots) was secured by considering a genotype-specific base temperature for stem elongation, *T*_bStE_, in the range of 8–10 °C independent of location. These experimentally founded values are much higher than those suggested for the variety Jorr (2–7.6 °C) (Martin and Stephens, 2008). The discrepancy between these base temperatures for shoot extension is probably due to a different interpretation of this parameter. In contrast to base temperature within a linear function of stem elongation rate and air temperature (Martin and Stephens, 2008), LUCASS used *T*_bStE_ to switch carbon allocation from ‘leaves only’ (*T*_*B*_) to ‘leaves+stems’, with stem elongation mainly depending on day length.

LAI values between 2 and 4 were sufficient to reach a high biomass yield (Jing *et al.*, 2012), suggesting that, with the exception of Endurance, all varieties were source limited during the first year of regrowth (Fig. 4 and Supplementary Fig. S3 at *JXB* online). As LUCASS simulated the seasonal dynamics of carbohydrate reserves explicitly, the reserve pools balanced the seasonal fluctuations in carbon availability. Overall, the agreement between measured and modelled sink indicators was good across the validation datasets (Table 3), even for stem numbers, although the dynamic of stem numbers was less well represented (Fig. 4). According to our simulations, the stem as the major sink accounted for 75–97% of the yield variance, variation of stool weights (Fig. 4; 47–95%), and plausible values for root dry matter. Furthermore, 94 and 80% of the yield variance was captured across cross-validation at ROTH and independent data sets (LARS and ROTH), respectively.

The necessity to define site-specific initial bud and stem numbers to compensate for environmental and management (coppicing) effects shows the need for a more mechanistic description of the coppicing response. Stored carbohydrates in the reserve pools are essential for the initial growth of perennials (Philippot, 1996), and the available evidence (Cunniff *et al.*, 2014, 2015) would allow implementation of a functional relationship between reserve availability and stem/bud numbers to describe regrowth (Bullard *et al.*, 2002b; Tharakan *et al.*, 2008).

Allocation to BGB was a limiting factor for development of AGB during crop establishment and can be considered as one cause for low yield during the first rotation. Water limitation caused a further significant reduction of AGB in favour of BGB at ROTH, and LUCASS simulated both limitations adequately. The varieties showed different responses towards WS from a very sensitive Endurance to an almost tolerant Resolution (Bonneau, 2004; Savage and Cavender-Bares, 2011; Larsen *et al.*, 2014). New evidence from specific experiments with potential- and limited-water treatments will follow in future.

From this analysis we suggest that the LUCASS model can be used first to accelerate the selection and optimization of genotypes in breeding programmes, and secondly to predict the site-specific yields of different SRC willow genotypes. Although details of the budburst modelling are still in progress, the current model can also be used to explore climate and management scenarios for the production of biomass resources in the bioeconomy. The ability of LUCASS to simulate allocation to BGB (stool, and coarse and fine roots) also helps to quantify the ecosystem functions of regrowth, soil-specific resource capture, and the carbon balance. Ongoing work will also provide more details on the sustainability of canopy and rooting phenotypes in different hydrological and agrometeorological situations.

## Supplementary data

Supplementary data are available at *JXB* online.

Model equations S1–S7.

**Fig. S1.** Canopy (1) and leaf (2) phenotypes for open, narrow-leaved Tora (A) and closed, broad-leaved Endurance (B).

**Fig. S2.** Global solar radiation (- -), air temperature (––) and precipitation (filled bar) at Rothamsted (A) and Aberystwyth (B) during the experiment (2010–13).

**Fig. S3.** Heat map from sensitivity analysis displaying the average response strength (*μ*) estimated using the Morris method, run for all varieties at both sites, Harpenden (ROTH) and Aberystwyth (ABER) with weather data for first (R1, 2010–11) and second rotation (R2, 2012–13).

**Fig. S4.** Observed (filled symbols) and simulated (solid line) leaf area index (LAI), canopy height, stem number and accumulated stem (AGB) and stool (BGB) biomass of Resolution (K–O) and Terra Nova (P–T) grown at Rothamsted over two consecutive 2-year rotations (2010–2011; 2012–2013).

**Table S1.** Physical characteristics of the soil in three sites using the Soil Classification System for England and Wales: Harpenden (ROTH), Aberystwyth (ABER) and Long Ashton Research Station (LARS).

**Table S2.** Cumulative annual precipitation, radiation, and average minimum and maximum temperature (2010–2013) at the two sites (ROTH and ABER).

**Table S3.** Optimized values of the parameters *T*_C_ and *C*_r_ of the chilling model for each willow variety.

**Table S4.** Results of the sensitivity analysis for ROTH and ABER simulated under potential (NWS) and water-limited (WS) production for (a) the first (2010–2011) and (b) the second (2012–2013) coppice rotation.

**Table S5.** Parameter values for the stem height/diameter relationship for the four studied varieties (Endurance, Resolution, Terra Nova, and Tora) and the two dedicated trials (ROTH and ABER).

Supplementary Data
