# Supplementary material for: Development of a sink–source interaction model for the growth of short-rotation coppice willow and in silico exploration of genotype×environment effects
Source: J Exp Bot. 2015 Dec 10;67(3):961–77. doi: 10.1093/jxb/erv507 (PMC4737082; doi:10.1093/jxb/erv507)
Supplement: Supplementary Data [file supp_67_3_961__index.html]

Development of a sink–source interaction model for the growth of short-rotation coppice willow and in silico exploration of genotype×environment effects — Development of a sink–source interaction model for the growth of short-rotation coppice willow and in silico exploration of genotype×environment effects — Supplementary Data 

# Development of a sink–source interaction model for the growth of short-rotation coppice willow and *in silico* exploration of genotype×environment effects

## Supplementary Data

Data files

- Supplementary\_figures\_S1\_S4\_Tables\_S1\_S5\_Equations.pdf - Supplementary Data
