# Supplementary material for: Development of a sink–source interaction model for the growth of short-rotation coppice willow and in silico exploration of genotype×environment effects
Source: J Exp Bot. 2015 Dec 10;67(3):961–77. doi: 10.1093/jxb/erv507 (PMC4737082; doi:10.1093/jxb/erv507)
Supplement: Supplementary Data [file supp_erv507_Supplementary_figures_S1_S4_Tables_S1_S5_Equations.pdf]

Supplement material

Essential model equations

Budburst model

$$C_u = \frac{-1}{1+\exp\left(a\left(T_{avr}-T_C\right)^2+b\left(T_{avr}-T_C\right)\right)}; \qquad C_a = \frac{1}{1+\exp\left(c\left(T_{avr}-T_C\right)\right)} \qquad \text{(S1)}$$

Leaf emergence and extension rate

The leaf emergence rate (*LfEmRt*) is computed with a deterministic model for leaf emergence in willow proposed by (Porter *et al.*, 1993) with *Doy* being the day of the year and *Dbf* the day of budburst, that is determined by the chilling model.

$$LfEmRt = \exp\left(-k \times (Doy - Dbf)\right) \text{ when } Doy > Dbf \qquad \text{(S2a)}$$

Porter's model was modified, to account for the influence of photoperiod (*fdayL*), water stress (k_{ws}) and level of reserves (*ResL*) on leaf emergence, as follows

$$LfEmRt = LfEmRt \times fdayL \times k_{ws} \times ResL \qquad \text{(S2b)}$$

$$k_{ws} = \frac{2}{1+\exp\left(-WSP \times \theta_r\right)} - 1 \qquad \text{(S2c)}$$

with θ_ρ being the relative soil water content and *WSP* the water stress parameter.

The leaf extension rate (*Extn_Lf*) is considered as dependent on day length (*DayL*), average temperature (T_{avr}) and plant age (f_{age}; (Robinson *et al.*, 2004)):

$$Extn\_Lf = DayL\left(m_{LER} \times \min\left(20, T_{avr}\right) - c_{LER}\right) \times f_{age} \times k_{ws} \qquad \text{(S3)}$$

where m_{LER} is the linear leaf extension rate (LER) coefficient and c_{LER} is the constant LER coefficient.

Leaf area calculation

The total daily potential leaf area (*LAIGrPt*) is defined as:

$$LAIGrPt = (NLf + TNBr \times NL_{Br}) \times (1 - LAI / MaxLAI) \times L_{\mathrm{fShp}} \times L_{fWth} \times Extn\_Lf \qquad \text{(S4)}$$

where *NLf* is the number of leaves per m^2, *TNBr* is total number of branches per m^2, *NfBr* is the number of leaves per branch, *MaxLAI* is the maximum leaf area per m^2, L_{fShp} is the leaf shape factor, L_{fWth} is the leaf width and finally *Extn-Lf* is the leaf extension rate.

Stem elongation

The potential stem elongation (*StExtPt*) depends on day-length and average day temperature (Powers *et al.*, 2006). In particular, stems extend according to a linear function of the day-length multiplied by a Heaviside function of the temperature (*DU*). Stems elongate in accordance to the hours of daylight, but the growth stops if the temperature is less than a minimum threshold. Stem elongation is affected by water stress. The model is as follows:

$$StExtPt = \max\left(0, \left(m_{SER} \times \left(DayL + dl_{0SER}\right)\right) \times DU\right) \times k_{ws} \tag{S5a}$$

$$\text{with} \quad DU = \max\left(0, \frac{T_{avr} - T_{base}}{\left|T_{avr} - T_{base}\right|}\right) \tag{S5b}$$

where DU is the developmental switch, which is 1 if T_{avr} is greater than a base temperature for stem elongation T_{base} otherwise is 0, m_{SER} is the slope of the stem elongation rate (SER) and dl_{0SER} is the minimum day length allowing stems to elongate.

Stem volume and stem weight

The net growth in the potential stem weight is based on the potential stem height. The diameter is computed as function of the newly updated stem height:

$$Diam = m_{DH}{}' \ (HStem + StExtPt) + h_{0DH} \tag{S6}$$

where m_{DH} is the slope in the relationship diameter/height, h_{0DH} is the intersect in the relationship diameter/height, which is the smaller height with a measurable diameter, and *HStem* is the stem height. Stems start thickening only after reaching a minimum height. Before that, the diameter equals the minimum measurable unit (1 mm).

The potential stem biomass (or dry matter (DM)) is computed as stem volume multiplied by the specific stem weight (ρ_{St}):

$$SDMCurr = \rho_{St} \times \left(\frac{\pi}{4} \times Diam^2 \times StExtPt \times TNSt\right) \times \eta_{St} \ . \tag{S7}$$

The daily-produced woody biomass is reduced by a multiplier or shape factor (η_{St}) that accounts for the stem deviation from a perfect cylinder.

Supplemental figures

Figure S1: Canopy (1) and leaf (2) phenotypes for open, narrow-leaved Tora (A) and closed, broad-leaved Endurance (B)

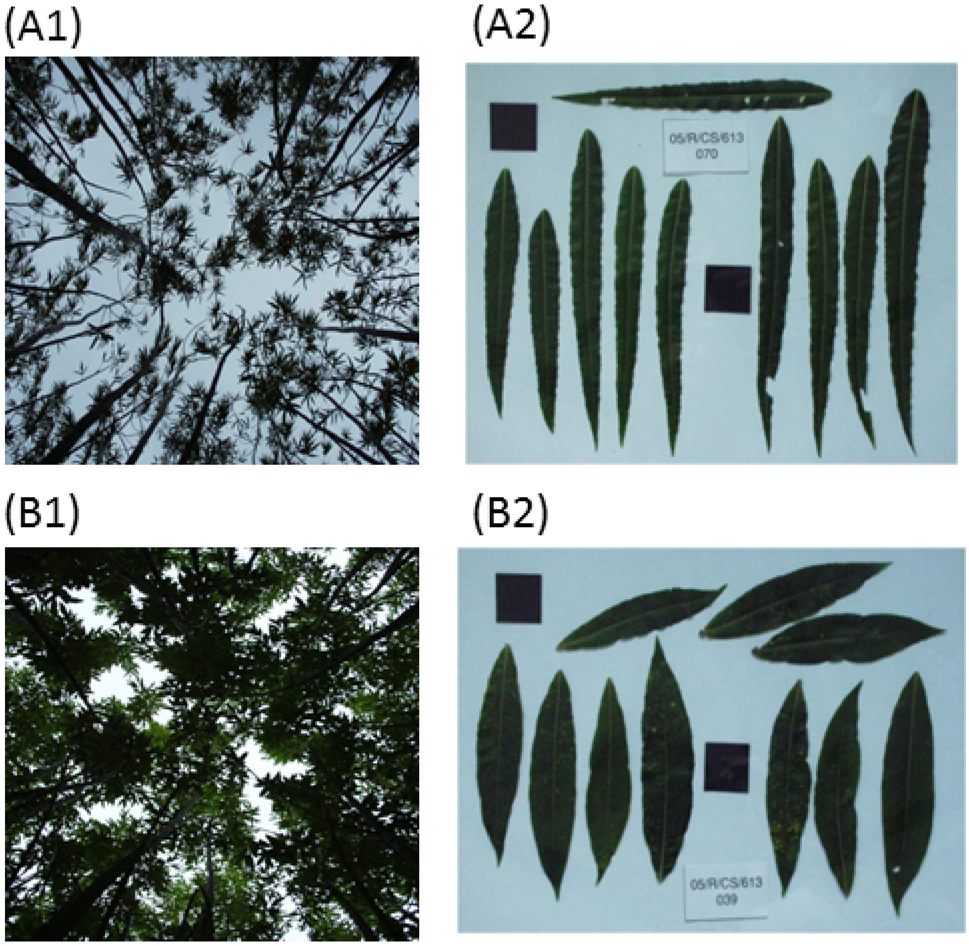

Figure S2: Global solar radiation (- -), air temperature (—) and precipitation (filled bar) at Rothamsted (A) and Aberystwyth (B)

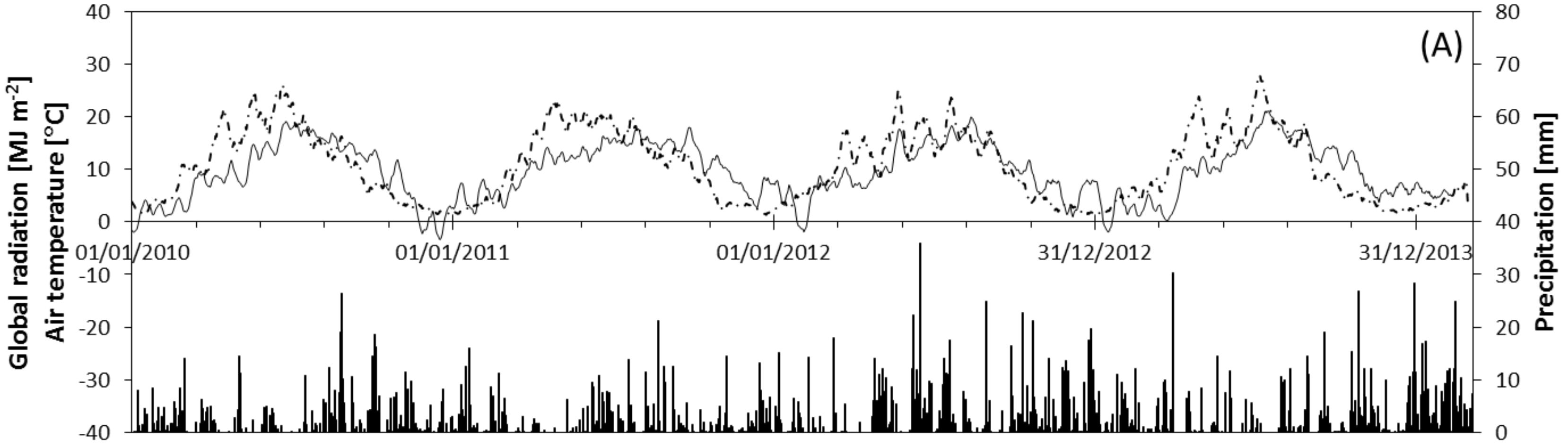

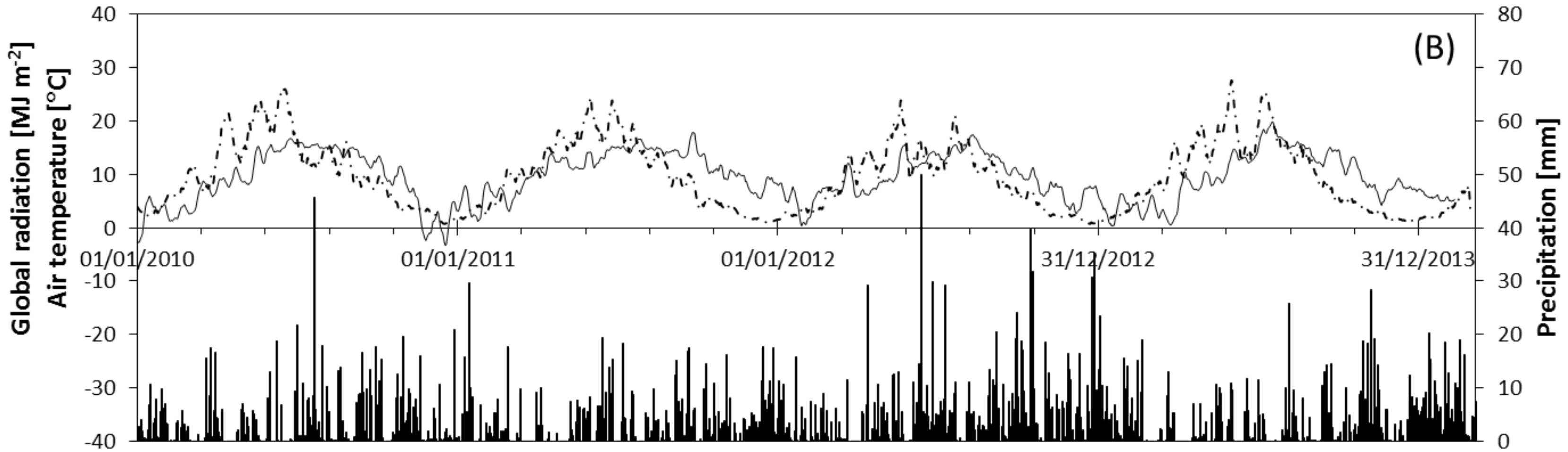

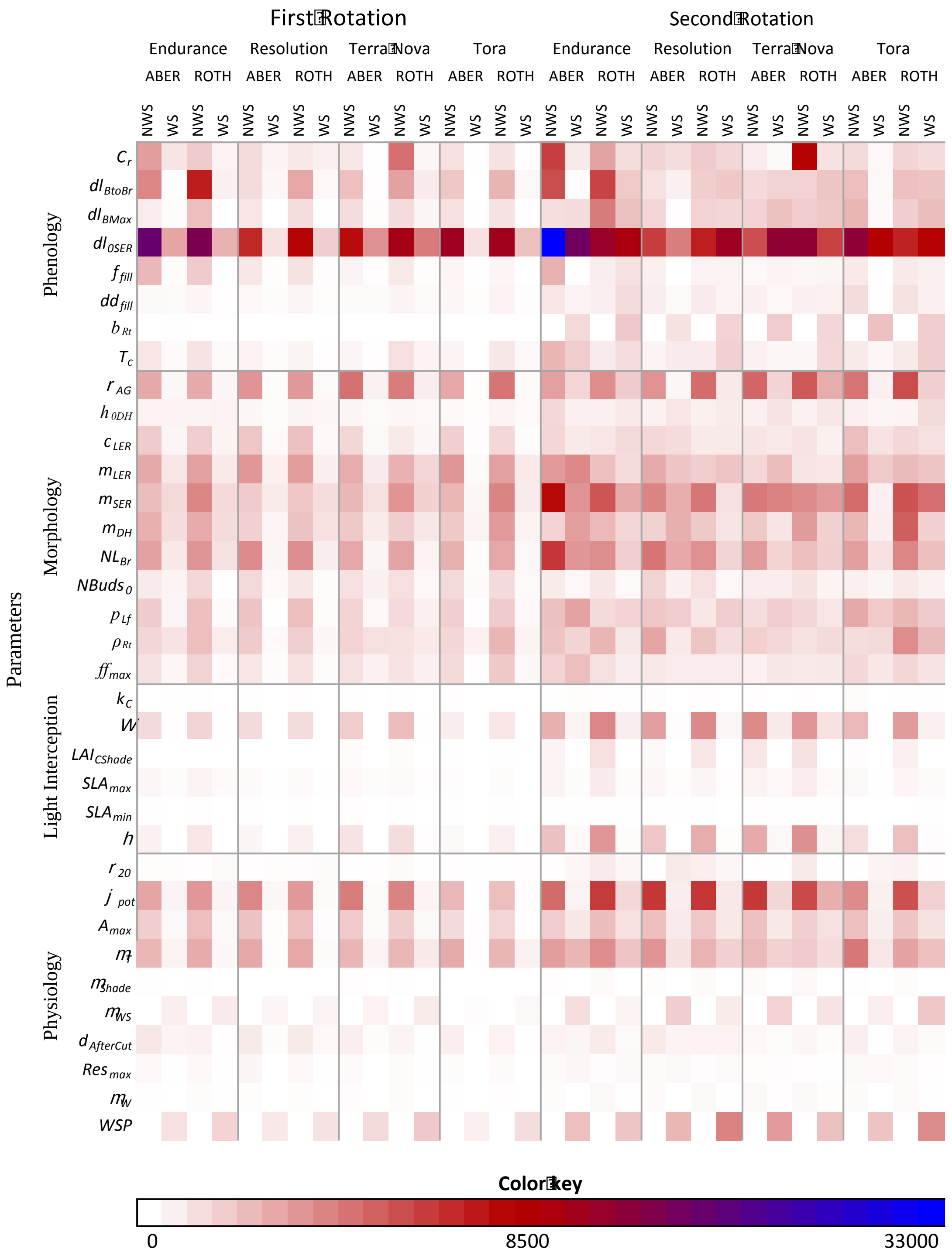

Figure S3: Heat map from sensitivity analysis displaying the average response strength (μ) estimated using the Morris' method, run for all varieties at both sites, Harpenden (ROTH) and Aberystwyth (ABER) with weather data for first (R1, 2010-11) and second rotation (R2, 2012-13). Simulations were done in absence of water stress (NWS) and under actual water stress (WS).

Figure S4: Observed (filled symbols) and simulated (solid line) leaf area index (LAI), canopy height, stem number and accumulated stem (AGB) and stool (BGB) biomass of Resolution (K-O) and Terra Nova (P-T) grown at Rothamsted over two consecutive 2-year rotations (2010-11; 2012-13). The error bars represent the standard deviations of the experimental values (n=4)

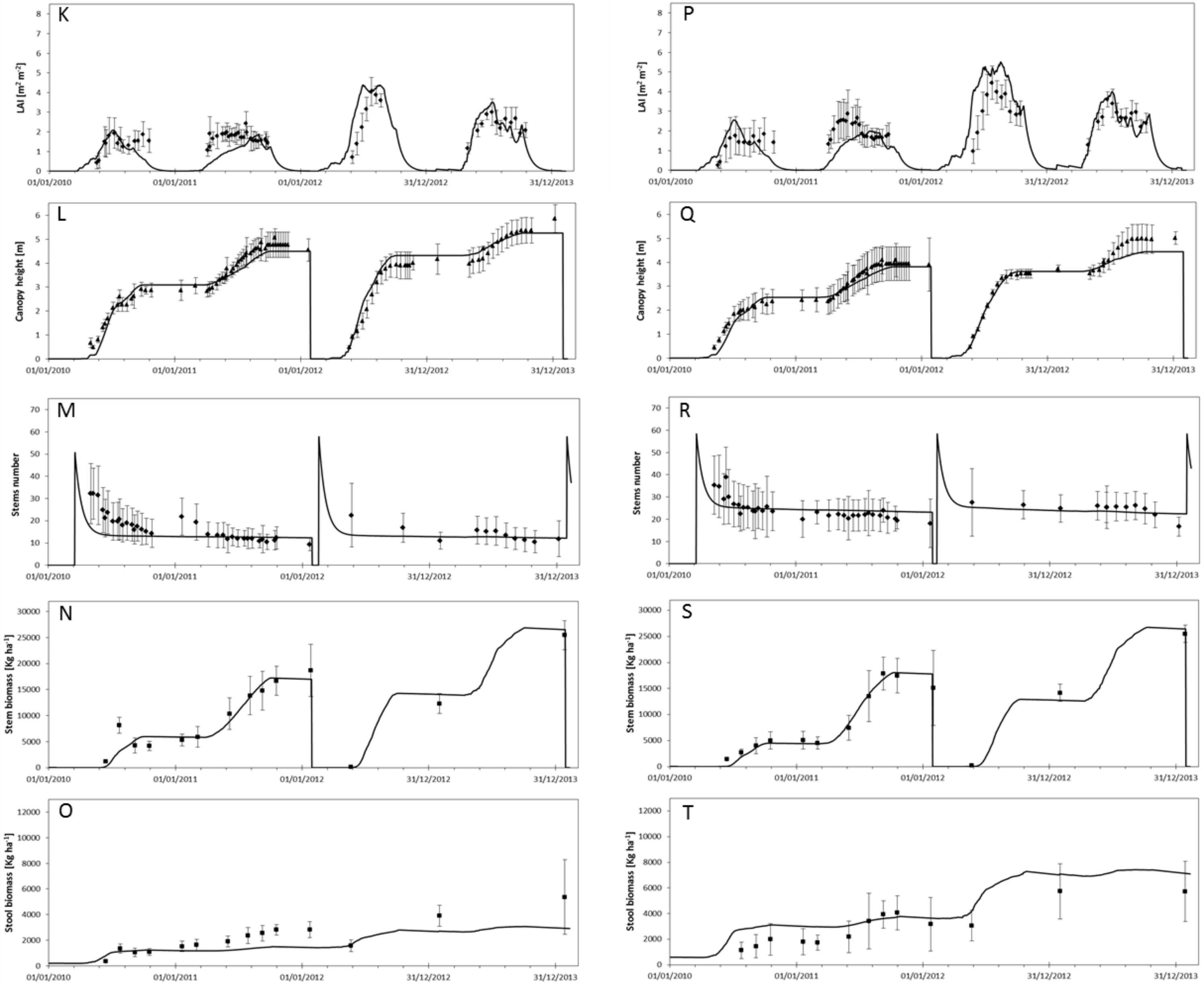

Supplemental Tables

Table S1: Physical characteristics of the soil in three sites using Soil Classification System for England and Wales: Harpenden (ROTH), Aberystwyth (ABER) and Long Ashton Research Station (LARS); soil depth, bulk density, soil texture (sand, silt and clay), soil organic carbon (SOC), volumetric water content at field capacity θ_{fc} and permanent wilting point θ_{pwp}, available water capacity (AWC) in the profile derived using the Hypres pedotransfer function (Wösten *et al.*, 1999).

**Site**	**Soil series**	**Soil depth** (m)	**Bulk density** Mg m^{-3}	**Soil texture (%)**			**SOC %**	θ_{fc} - θ_{pwp}	**AWC (mm)**
				**Sand**	**Silt**	**Clay**			
ROTH	Batcombe-Carsten	1.00	1.62	13	62	25	0.73	0.36 – 0.18	180
ABER	Denbigh	0.55	1.26	41	51	8	0.68	0.40 – 0.07	182
LARS	Greinton – Worcester	0.95	1.37	33	40	27	1.57	0.38 – 0.17	209

Table S2 Cumulative annual precipitation, radiation, and average minimum and maximum temperature (2010-2013) and the two sites (ROTH and ABER)

Year	ABER				ROTH			
	T MAX (°C)	T MIN (°C)	RAIN (mm)	RAD (MJ/m²/d)	T MAX (°C)	T MIN (°C)	RAIN (mm)	RAD (MJ/m²/d)
2010	12.7	5.1	892	10.3	12.7	5.3	644.2	10.8
2011	14.3	7.6	813.4	9.8	14.7	6.9	571.5	11
2012	12.9	7.5	1152.8	8.6	13.5	5.9	1059.2	10.5
2013	13.1	7.2	873.5	9.8	13.2	5.8	748.9	10.6

Table S3: Optimised values of the parameters T_C and C_r of the chilling model (1) for each willow variety.

**Variety**	T_C	C_r
**Endurance**	6.4	17.7
**Resolution**	6.6	18.6
**Terra Nova**	5.6	18.0
**Tora**	5.7	18.1

Table S4: Results of the sensitivity analysis for ROTH and ABER simulated under potential (NWS) and water-limited (WS) production for (a1, a2) the first (R1, 2010-11) and (b1, b2) the second coppice rotation (R2, 2012-13)

(a1)

ROTH 2010-11											
**Potential/NWS conditions**						**Water-limited conditions**					
**Parameter**	**Process**	**μ**	**SD**	**σ**	**SD**	**Parameter**	**Process**	**μ**	**SD**	**σ**	**SD**
dl_{0SER}	Phenology	11.60	3.37	7.62	1.73	dl_{0SER}	Phenology	2.70	1.22	1.95	0.45
dl_{BtoBr}	Phenology	4.05	2.35	4.02	1.46	WSP	Physiology	1.35	0.37	0.91	0.21
ρ_{AG}	Morphology	3.83	0.83	1.71	0.72	m_{SER}	Morphology	1.17	0.37	0.98	0.61
m_{SER}	Morphology	3.41	1.04	1.70	0.32	m_{LER}	Morphology	0.87	0.37	1.16	0.44
NL_{Br}	Morphology	3.33	0.39	3.17	0.67	m_{DH}	Morphology	0.86	0.34	0.85	0.51
φ_{pot}	Physiology	3.32	0.83	1.14	0.23	NL_{Br}	Morphology	0.58	0.33	0.63	0.39
m_{LER}	Morphology	3.05	0.32	1.81	0.17	ρ_{Rt}	Morphology	0.54	0.20	0.55	0.34
μ_T	Physiology	2.67	0.28	1.10	0.30	μ_{WS}	Physiology	0.50	0.22	0.72	0.47
m_{DH}	Morphology	2.48	0.79	1.44	0.44						
C_r	Phenology	2.09	1.90	3.86	4.57						
A_{max}	Physiology	1.85	0.37	0.81	0.31						
p_{Lf}	Morphology	1.84	0.44	0.78	0.33						
ρ_{Rt}	Morphology	1.83	0.65	1.09	0.56						
c_{LER}	Morphology	1.46	0.57	0.89	0.13						
Ω	L.I.	1.44	0.56	0.86	0.41						
dl_{BMax}	Phenology	1.38	0.55	1.33	0.27						
ff_{max}	Morphology	1.33	0.41	0.67	0.35						
$NBuds_0$	Morphology	1.13	0.24	0.94	0.38						
f_{fill}	Phenology	0.95	0.60	1.09	1.32						
T_c	Phenology	0.82	0.28	0.69	0.32						
η	L.I.	0.77	0.30	0.62	0.22						
$d_{AfterCut}$	Physiology	0.51	0.15	0.27	0.08						

(a2)

ABER 2010-2011												
**Potential/no water stress conditions**							**Water-limited conditions**					
**Parameter**	**Process**	**µ**	**SD**	**σ**	**SD**		**Parameter**	**Process**	**µ**	**SD**	**σ**	**SD**
dl_{0SER}	Phenology	11.59	5.43	6.05	1.23		dl_{0SER}	Phenology	2.18	1.38	1.84	1.17
ρ_{AG}	Morphology	3.54	0.87	1.08	0.43		WSP	Physiology	0.91	0.31	0.56	0.25
φ_{pot}	Physiology	3.45	0.91	1.14	0.09		m_{SER}	Morphology	0.83	0.43	0.78	0.60
NL_{Br}	Morphology	3.16	0.54	2.23	0.70		ρ_{Rt}	Morphology	0.69	0.40	0.66	0.40
m_{LER}	Morphology	3.15	0.40	1.39	0.14		m_{DH}	Morphology	0.64	0.37	0.68	0.46
μ_T	Physiology	2.68	0.26	0.96	0.32		m_{LER}	Morphology	0.57	0.27	0.61	0.51
dl_{BtoBr}	Phenology	2.32	1.23	2.84	1.55							
m_{SER}	Morphology	2.21	0.32	1.61	0.47							
m_{DH}	Morphology	1.98	0.46	0.87	0.19							
p_{Lf}	Morphology	1.73	0.22	0.57	0.33							
A_{max}	Physiology	1.70	0.33	0.56	0.22							
c_{LER}	Morphology	1.67	0.23	0.82	0.09							
C_r	Phenology	1.58	1.14	2.06	1.99							
ρ_{Rt}	Morphology	1.50	0.17	0.76	0.28							
Ω	L.I.	1.21	0.46	0.71	0.24							
$NBuds_0$	Morphology	1.08	0.28	0.87	0.46							
ff_{max}	Morphology	1.03	0.15	0.30	0.14							
f_{fill}	Phenology	0.98	0.96	0.94	1.07							
dl_{BMax}	Phenology	0.94	0.23	0.93	0.49							
$d_{AfterCut}$	Physiology	0.67	0.11	0.40	0.28							
η	L.I.	0.51	0.31	0.48	0.13							
T_c	Phenology	0.50	0.23	0.49	0.31							

*L.I.: Light interception

(b1)

ROTH 2012-13											
**Potential/no water stress conditions**						**Water-limited conditions**					
**Parameter**	**Process**	**μ**	**SD**	**σ**	**SD**	**Parameter**	**Process**	**μ**	**SD**	**σ**	**SD**
dl_{0SER}	Phenology	10.25	3.28	7.49	3.04	dl_{0SER}	Phenology	9.15	2.34	5.97	1.31
φ_{pot}	Physiology	6.31	0.40	2.77	0.45	m_{SER}	Morphology	3.03	1.55	2.22	1.36
ρ_{AG}	Morphology	5.07	0.93	1.60	0.43	WSP	Physiology	3.01	1.14	2.11	0.89
m_{SER}	Morphology	4.98	0.92	2.47	0.12	dl_{BtoBr}	Phenology	1.91	0.09	2.27	0.54
Ω	L.I.	3.72	0.32	1.68	0.29	dl_{BMax}	Phenology	1.89	0.38	1.94	0.79
C_r	Phenology	3.68	3.28	6.99	8.50	μ_T	Physiology	1.74	0.41	1.39	0.52
NL_{Br}	Morphology	3.45	0.86	5.36	1.02	ρ_{AG}	Morphology	1.73	0.83	0.96	0.18
m_{DH}	Morphology	3.23	1.57	3.19	3.65	NL_{Br}	Morphology	1.72	0.32	1.57	0.74
η	L.I.	3.05	0.75	1.53	0.14	b_{Rt}	Phenology	1.59	0.19	1.23	0.59
dl_{BtoBr}	Phenology	2.89	2.27	2.72	1.86	φ_{pot}	Physiology	1.59	0.81	0.96	0.11
μ_T	Physiology	2.82	0.87	2.56	0.83	m_{LER}	Morphology	1.50	0.55	1.08	0.41
ρ_{Rt}	Morphology	2.34	1.17	3.28	2.82	p_{Lf}	Morphology	1.47	0.40	1.27	0.64
dl_{BMax}	Phenology	2.33	1.40	1.83	1.01	T_c	Phenology	1.43	0.23	1.26	0.58
A_{max}	Physiology	1.90	0.25	1.25	0.25	m_{DH}	Morphology	1.37	0.31	1.04	0.42
m_{LER}	Morphology	1.76	0.61	1.61	0.47	ρ_{Rt}	Morphology	1.37	0.63	0.88	0.09
p_{Lf}	Morphology	1.47	0.68	1.37	0.78	C_r	Phenology	1.17	0.15	1.36	0.26
c_{LER}	Morphology	0.97	0.28	1.31	0.49	μ_{WS}	Physiology	0.99	0.65	0.89	0.53
ff_{max}	Morphology	0.96	0.33	0.84	0.51	A_{max}	Physiology	0.98	0.14	0.56	0.14
$NBuds_0$	Morphology	0.91	0.16	0.55	0.02	h_{0DH}	Morphology	0.90	0.27	0.93	0.36
LAI_{CShade}	L.I.	0.87	0.25	0.35	0.14	c_{LER}	Morphology	0.90	0.34	0.91	0.44
dd_{fill}	Phenology	0.71	0.22	0.62	0.36	ff_{max}	Morphology	0.74	0.15	0.89	0.34
ρ_{20}	Physiology	0.61	0.11	0.17	0.02	dd_{fill}	Phenology	0.66	0.34	0.74	0.34
T_c	Phenology	0.61	0.18	0.68	0.16	Ω	L.I.	0.63	0.30	0.37	0.18
f_{fill}	Phenology	0.53	0.19	0.42	0.21	f_{fill}	Phenology	0.59	0.30	0.86	0.93
SLA_{max}	L.I.	0.51	0.19	0.25	0.24						
h_{0DH}	Morphology	0.49	0.06	0.29	0.06						
$d_{AfterCut}$	Physiology	0.46	0.15	0.49	0.26						

*L.I.: Light interception

(b2)

ABER 2012-13												
**Potential/no water stress conditions**							**Water-limited conditions**					
**Parameter**	**Process**	**μ**	**SD**	**σ**	**SD**		**Parameter**	**Process**	**μ**	**SD**	**σ**	**SD**
dl_{0SER}	Phenology	14.89	12.82	9.18	5.65		dl_{0SER}	Phenology	11.10	5.91	6.38	1.37
φ_{pot}	Physiology	5.54	1.37	2.49	0.25		m_{SER}	Morphology	2.75	1.59	3.18	1.88
m_{SER}	Morphology	5.44	1.89	4.10	2.07		WSP	Physiology	2.51	0.64	2.57	0.58
NL_{Br}	Morphology	4.47	1.60	5.97	2.29		m_{LER}	Morphology	2.48	0.99	2.39	0.72
ρ_{AG}	Morphology	4.15	0.91	1.09	0.37		NL_{Br}	Morphology	2.25	1.16	2.44	1.59
μ_{T}	Physiology	3.44	0.90	2.41	0.77		p_{Lf}	Morphology	2.07	0.67	2.44	0.95
Ω	L.I.	3.03	0.69	1.38	0.35		m_{DH}	Morphology	1.86	1.32	2.06	1.63
m_{LER}	Morphology	2.70	0.95	2.12	0.53		b_{Rt}	Phenology	1.51	0.48	1.50	0.82
dl_{BtoBr}	Phenology	2.61	2.26	3.18	2.87		μ_{T}	Physiology	1.48	0.74	1.56	0.63
C_{r}	Phenology	2.45	2.68	3.63	4.54		μ_{WS}	Physiology	1.22	0.47	1.80	0.89
A_{max}	Physiology	2.02	0.21	0.99	0.15		ρ_{Rt}	Morphology	1.20	0.31	1.28	0.67
p_{Lf}	Morphology	2.00	0.72	1.58	0.52		ff_{max}	Morphology	1.12	0.72	1.35	0.97
η	L.I.	1.99	0.71	1.36	0.37		ρ_{AG}	Morphology	0.93	0.59	1.00	0.41
ρ_{Rt}	Morphology	1.93	0.80	2.29	1.29		dl_{BMax}	Phenology	0.92	0.96	0.88	0.79
m_{DH}	Morphology	1.83	0.42	1.36	0.43		c_{LER}	Morphology	0.91	0.24	1.08	0.41
dl_{BMax}	Phenology	1.63	0.60	1.80	0.74		T_{c}	Phenology	0.76	0.66	0.93	1.01
c_{LER}	Morphology	1.45	0.53	1.58	0.50		φ_{pot}	Physiology	0.74	0.42	1.03	0.25
f_{fill}	Phenology	1.04	1.05	1.07	1.20		A_{max}	Physiology	0.73	0.09	0.58	0.11
ff_{max}	Morphology	1.03	0.48	0.74	0.35		h_{0DH}	Morphology	0.67	0.28	0.76	0.28
T_{c}	Phenology	1.02	0.98	1.47	2.18		C_{r}	Phenology	0.60	0.42	0.80	0.68
$NBuds_{0}$	Morphology	0.83	0.41	0.75	0.84		dl_{BtoBr}	Phenology	0.60	0.61	0.78	0.82
dd_{fill}	Phenology	0.81	0.32	0.56	0.30							
h_{0DH}	Morphology	0.71	0.40	0.61	0.45							
$d_{AfterCut}$	Physiology	0.51	0.19	0.63	0.25							

Table S5: Parameter values for the stem height/diameter relationship, for the four studied varieties (Endurance, Resolution, Terra Nova and Tora) and the two dedicated trials (ROTH and ABER).

		ROTH				
**Variety**		**Coefficient**	**SE**	**t**	**p**	R^2
Endurance	$c_{D/H}$	-0.40	0.03	-12.7078	<0.0001	0.80
	$m_{D/H}$	5.47	0.11	49.8054	<0.0001	
Resolution	$c_{D/H}$	-0.32	0.03	-10.3539	<0.0001	0.96
	$m_{D/H}$	4.52	0.06	72.1593	<0.0001	
Terra Nova	$c_{D/H}$	-0.52	0.03	-18.8875	<0.0001	0.93
	$m_{D/H}$	5.39	0.09	59.6124	<0.0001	
Tora	$c_{D/H}$	-0.26	0.05	-5.5800	<0.0001	0.78
	$m_{D/H}$	4.67	0.11	41.1339	<0.0001	
		**ABER**				
Endurance	$c_{D/H}$	-0.56	0.06	-9.314	<0.0001	0.81
	$m_{D/H}$	7.12	0.17	41.3366	<0.0001	
Resolution	$c_{D/H}$	-0.40	0.10	-4.0806	<0.0001	0.80
	$m_{D/H}$	5.94	0.17	35.5307	<0.0001	
Terra Nova	$c_{D/H}$	-0.39	0.06	-7.0471	<0.0001	0.83
	$m_{D/H}$	6.12	0.14	44.8653	<0.0001	
Tora	$c_{D/H}$	-0.83	0.05	-16.7671	<0.0001	0.90
	$m_{D/H}$	7.06	0.13	55.9181	<0.0001	

References

Porter JR, Parfitt RI, Arnold GM. 1993. Leaf Demography in Willow Short-Rotation Coppice. *Biomass & Bioenergy* **5**, 325-336.
Powers SJ, Peacock L, Yap ML, Brain P. 2006. Simulated beetle defoliation on willow genotypes in mixture and monotype plantations. *Annals of Applied Biology* **148**, 27-38.
Robinson KM, Karp A, Taylor G. 2004. Defining leaf traits linked to yield in short-rotation coppice Salix. *Biomass & Bioenergy* **26**, 417-431.
Wösten JHM, Lilly A, Nemes A, Le Bas C. 1999. Development and use of a database of hydraulic properties of European soils. *Geoderma* **90**, 169-185.
